# Reactivation and long-term stabilization of the [NiFe] Hox hydrogenase of *Synechocystis* sp. PCC6803 by glutathione after oxygen exposure

**DOI:** 10.1016/j.jbc.2024.108086

**Published:** 2024-12-14

**Authors:** Merle Romig, Marie Eberwein, Darja Deobald, Andreas Schmid

**Affiliations:** 1 Department of Solar Materials Biotechnology, Helmholtz Centre for Environmental Research - UFZ GmbH, Leipzig, Germany; 2 Department of Molecular Environmental Biotechnology, Helmholtz Centre for Environmental Research - UFZ GmbH, Leipzig, Germany

**Keywords:** protein complex, redox regulation, photosynthesis, cyanobacteria, enzyme inactivation

## Abstract

Hydrogenases are key enzymes forming or consuming hydrogen. The inactivation of these transition metal biocatalysts with oxygen limits their biotechnological applications. Oxygen-sensitive hydrogenases are distinguished from oxygen-insensitive (tolerant) ones by their initial hydrogen turnover rates influenced by oxygen. Several hydrogenases, such as the oxygen-sensitive bidirectional [NiFe] Hox hydrogenase (Hox) of the unicellular cyanobacterium *Synechocystis* sp. PCC6803, are reactivated after oxygen-induced deactivation by redox mechanisms. In cyanobacteria, the glutathione (GSH) redox buffer majorly controls intracellular redox potentials. The relationship between Hox turnover rates and the redox potential in its natural reaction environment is not fully understood. We thus determined hydrogen oxidation rates as activities of Hox in cell-free extracts of *Synechocystis* using benzyl viologen as artificial electron acceptor. We found that GSH modulates Hox hydrogen oxidation rates under oxygen-free conditions. After oxygen exposure, it influences the maximal turnover rate and aids in the reactivation of Hox. Moreover, GSH stabilizes the long-term Hox activity under anoxic conditions and attenuates oxygen-induced deactivation of Hox in a concentration-dependent manner, probably by fostering reactivation. Conversely, oxidized GSH (GSSG) negatively affects Hox activity and oxygen insensitivity. Using Blue Native PAGE followed by mass spectrometry, we showed that oxygen affects Hox complex integrity. The *in silico* predicted structure of the Hox complex and complexome analyses reveal the formation of various Hox subcomplexes under different conditions. Our findings refine our current classification of oxygen–hydrogenase interactions beyond sensitive and insensitive, which is particularly important for understanding hydrogenase function under physiological conditions in future.

Redox enzymes play crucial roles in many metabolic pathways, including methanogenesis (1, 2), organohalide respiration (3), hydrogen (H_2_) metabolism (4, 5, 6), as well as nitrogen (7) and carbon dioxide fixation (8). These enzymes commonly bind different metallocofactors sensitive to oxygen, such as cofactor F_430_, cobalamin, iron-sulfur-molybdenum clusters, and diverse nickel-iron [NiFe] or nickel-iron-sulfur/selenium [NiFeS/Se] and iron-iron [FeFe] clusters. The sensitivity of these metal clusters is often due to oxidative damage (9, 10), generation of reactive oxygen species (11, 12), oxygen reacting with metal centers (12), and changes in the redox state and coordination environment of the metal active sites (13) upon exposure to oxygen, finally leading to enzyme inactivation.

Microorganisms have evolved diverse strategies to protect these cofactors from oxidative damage. Strictly anaerobic organisms, such as methanogenic archaea, protect oxygen-labile enzymes by inhabiting strictly anoxic environments, and adapting to anaerobic metabolism (2). Nitrogen-fixing *Rhizobia* synthesize cytosolic oxygen scavengers like leghemoglobin to remove oxygen (14, 15). ATP-dependent electron transferases, such as reductive activators of corrinoid enzymes, reactivate catalytically inactive cob(II)alamin to cob(I)alamin in corrinoid-dependent proteins (16). Filamentous nitrogen-fixing cyanobacteria, such as *Anabena* sp. PCC7120 (17) and *Nostoc punctiforme* (18), spatially separate oxygen-producing photosynthesis from oxygen-sensitive nitrogen-fixing enzymes by differentiating vegetative cells into heterocysts (7, 19). Unicellular cyanobacteria *Synechococcus* (20) and *Cyanothece* sp. ATCC 51142 (7) temporally separated expression of photosynthetic genes during light, while oxygen-sensitive enzymes are produced during dark phases (7, 20). Redox buffer systems are also crucial components of cellular defense against oxidative stress, providing essential protection for oxygen-sensitive enzymes (21, 22). Glutathione (GSH), the most abundant low molecular weight thiol in many organisms (23), fulfills antioxidant functions (24). In *Synechocystis* sp. PCC6803 (*Synechocystis*), GSH or its precursor γ-glutamyl-cysteine are indispensable for growth (25, 26) and is present in intracellular concentrations ranging from 2.2 to 4.8 mM, depending on light intensity, wavelength, and carbon source during growth (26, 27). In the cytoplasm, GSH remains predominantly in its reduced form (26, 28) and the concentration of GSSG, its oxidized form, is often below 5% (26). Under stress conditions, such as an excess of reactive oxygen species, reduced GSH dimerizes to oxidized GSSG, forming a disulfide bridge (29). Glutathionylation of cysteine residues in proteins is also induced by oxygen stress, protecting proteins against oxygen-induced damage (23), as shown by mass spectrometry (30).

Hydrogenases, present in all three domains of life including lower eukaryotes, archaea, and bacteria (31), are transition metal biocatalysts, catalyzing both the oxidation and formation of H_2_ (32). They are categorized as [NiFe], [FeFe], and [Fe] hydrogenases based on their active center metal ions (33). Although [FeFe] hydrogenases are more efficient in H_2_ production, their high sensitivity towards oxygen, often leading to irreversible enzyme deactivation, limits their biotechnological use (33). Conversely, [NiFe] hydrogenases typically favor H_2_ oxidation and exhibit lower turnover numbers for H_2_ production than [FeFe] hydrogenases (33). Although generally regarded as oxygen-sensitive, several [NiFe] hydrogenases, such as the H_2_-sensing regulatory [NiFe] hydrogenase in the Knallgas bacterium *Cupriavidus necator*, demonstrate oxygen insensitivity (34), and oxygen-induced deactivation is often reversible (35). Standard [NiFe] hydrogenases exhibit two major inactive states: (*i*) Ni-A, referred to as “unready” state, reactivating slowly and binding a (hydro)peroxo species in the active site and (*ii*) Ni-B, referred to as the “ready” state, reactivating quickly and containing a (hydr)oxo group in the active site (36). In standard [NiFe] hydrogenases, nickel can exist in eight major states (Ni-A, Ni-B, Ni-C, Ni-R, Ni-SI_a_, Ni-SU, and Ni-SI_r_), detectable *via* FTIR spectroscopy, while only three of these states (Ni-A, Ni-B, and Ni-C) are EPR-active.

The unicellular cyanobacterium *Synechocystis* is mainly gaining attention for its photoproduction of H_2_ *via* a bidirectional [NiFe] hydrogenase (Hox) complex (37, 38). This complex operates as a dimerized heteropentamer, Hox(HYEUF)_2_ (39), encoded by an octacistronic *hox* operon (40, 41, 42). While oxygen sensitivity of Hox complex poses a significant challenge for applying *Synechocystis* as a chassis for oxygenic photoproduction of H_2_, previous studies showed that Hox from *Synechocystis* has a catalytic bias towards H_2_ formation (43). In addition, it retains 25 to 50% of its maximal H_2_ forming activity in the presence of 1% oxygen (43) with reactivation occurring within 1 to 2 min after oxygen exposure under anoxic conditions *in vivo* (37). *In vitro*, Hox reactivation is redox-dependent and facilitated by reduced electron donors such as NAD(P)H (44). Ni-B-like, Ni-SI_a_-like, Ni-C, and Ni-R states, but not the catalytically inactive Ni-A, Ni-SU, and Ni-SI_r_ states, have been detected by FTIR spectroscopy in the Hox complex of *Synechocystis*. Notably, the Ni-B-like state observed in Hox did not produce a corresponding EPR signal (45, 46). Contrary to the FTIR results, protein film electrochemistry (PFE) analysis of immobilized Hox revealed that two distinct inactive states are formed upon oxygen exposure, differing from the classical Ni-A and Ni-B states. While a major population was reactivated at low potentials below −563 mV, a minor population of an oxidized, inactive state of Hox was reactivated at higher potentials between −200 and −100 mV, showcasing unique redox properties of Hox from *Synechocystis* (46). Although PFE is a powerful and popular method for studying the reactivation of [NiFe] hydrogenases, it has limitations such as heterogeneous protein distribution and orientation on the electrode, the influence of the electrode’s local environment, which differs from natural cellular conditions, as well as kinetic complications due to complex electron transfer processes.

The relationship between Hox turnover rates and the redox potential in its natural environment is not well understood and is a focus of this study. Considering the role of the GSH/GSSG redox pair in reflecting cellular electron availability (47, 48, 49) and in modulating intracellular redox potentials (50), we suggest that GSH regulates Hox activity in *Synechocystis.* To assess the influence of GSH on Hox activity and Hox complex integrity, we conducted complexome analyses of crude extracts from *Synechocystis* after oxygen exposure and GSH treatment using Blue Native PAGE (BN-PAGE) and protein mass spectrometry. Additionally, we performed comprehensive kinetic studies *via* photometric enzyme activity assays using benzyl viologen (BV) as an electron acceptor and H_2_ as an electron donor. We show that Hox activity in *Synechocystis* is modulated by GSH, particularly at cytosolic concentrations. Our findings reveal a favorable impact of GSH on maximal and long-term Hox activities in samples exposed and not exposed to oxygen. Notably, there was a substantial increase in the reactivation of Hox activity in oxygen-exposed crude extracts treated with GSH. This effect was concentration-dependent. Conversely, oxidized GSSG had a detrimental effect on Hox reactivation. Our data underscore the pivotal role of GSH as a key component in the cellular redox buffer system of *Synechocystis*, stabilizing Hox activity both *in vivo* and *in vitro* revealing different effects on initial *versus* long term activities. These findings refine our understanding of interactions between oxygen and [NiFe] hydrogenases beyond the binary classification of sensitive and insensitive, which might be crucial for understanding [NiFe] hydrogenase function under physiological conditions.

## Results

In this study, we elucidate the influence of oxygen exposure and GSH treatment on Hox hydrogenase activity and protein complex integrity. First, we conducted endpoint activity measurements and complexome analyses of Hox in crude extracts derived from *Synechocystis* using BN-PAGE. Recognizing the limitations of endpoint activity determinations in deducing enzyme kinetics, quantitative photometric measurements of hydrogenase activity under diverse conditions, as outlined in subsequent sections, were performed. Absolute initial, maximal, and long-term hydrogenase activities were calculated using continuous photometric monitoring, with activities normalized to protein content. To ensure consistency, cells used for the photometric assays were always harvested at the same growth phase (8 days of cultivation under nitrogen-limited conditions). However, absolute Hox activities in samples not exposed to oxygen varied across extracts (Table S1), likely due to differences in Hox hydrogenase abundance. To address this variability, we calculated relative Hox activities by comparing the initial, maximal, or long-term activities of treated samples (*e.g*., those exposed to oxygen) with untreated controls. This approach enabled us to determine the degree of inactivation or reactivation for Hox hydrogenase, providing insights into the kinetics of the enzyme’s reactivation mechanism. To minimize the effects of autotrophic/mixotrophic growth conditions on the intracellular oxygenic environment in *Synechocystis*, which can introduce variability in Hox hydrogenase and is controlled by homoeostasis, we consistently used cell-free crude extracts from the same culturing conditions (8-days cultivation under nitrogen-limiting conditions) in our photometric activity assays. This deliberate approach ensured the Hox-containing protein fractions were of consistent quality, allowing us to reliably study how GSH/GSSG affects Hox activity and complex integrity *in vitro* and putatively in the cytoplasm, as close to the natural reaction environment of the Hox hydrogenase as possible.

### *In silico* structure of the dodecameric Hox hydrogenase complex

**Figure 1:**
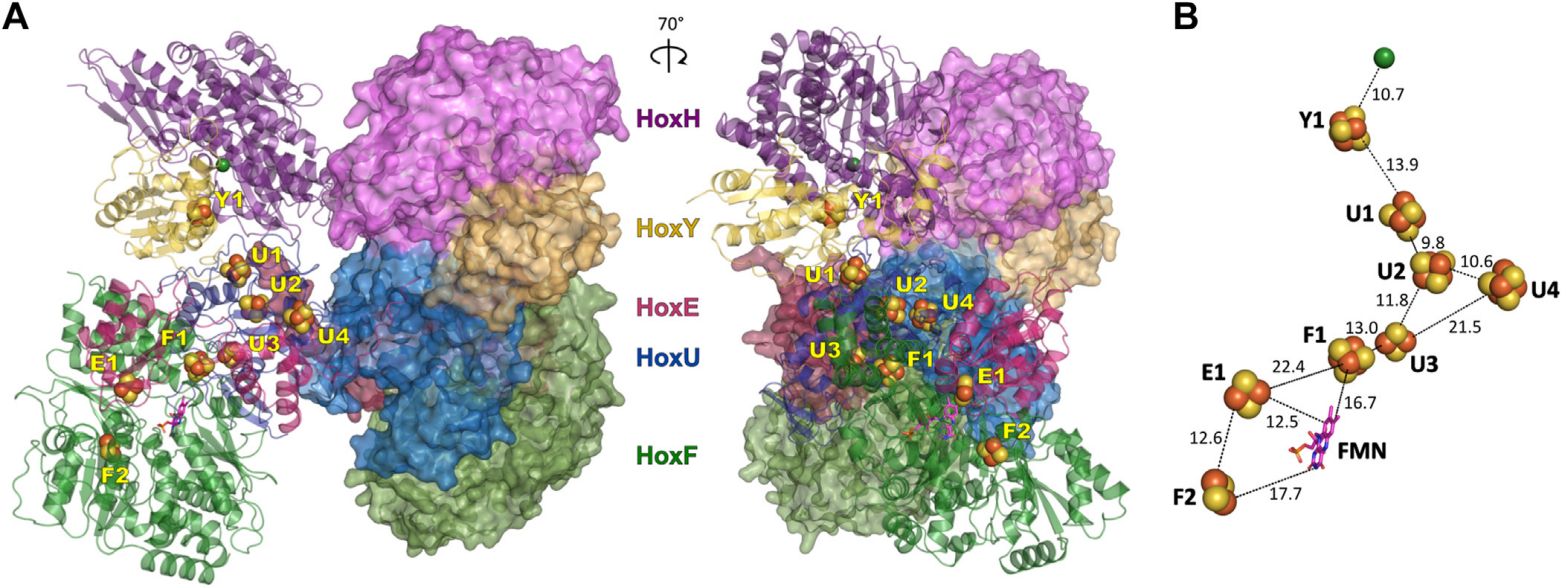
***In silico* predicted structure of the Hox(HYEUF)**_**2**_**complex from *Synechocystis* sp. PCC6803, calculated using AlphaFold 2.***A*, the *left* panel shows one heteropentamer of HoxHYEUF in cartoon representation (*left* side), with predicted iron-sulfur clusters (Y1 to F2, *yellow*-*orange* spheres) and FMN (*pink*, stick representation), while the second HoxHYEUF pentamer (*right* side) is shown in surface representation. Both pentamers dimerize *via* HoxH, HoxU, and HoxE. The *right* panel shows the Hox(HYEUF)_2_ structure rotated 70° to the right around the y-axis. *B*, the iron-sulfur cofactors and FMN forming an electron-conducting ‘wire’ are presented. Distances between the cofactors are given in angstroms (Å).

Using AlphaFold 2, we calculated the protein structure of the dodecameric Hox(HYEUF)_2_ complex, achieving a confidence score of 92.9 (Fig. 1). Although conducting cross-linking experiments could provide valuable additional insights into the three-dimensional structure of Hox, these experiments, particularly for low-abundance proteins like Hox from *Synechocystis*, are challenging and not straightforward (51). In this study, however, our focus was not on in-depth structural analysis but rather on using the predicted Hox structure to support observations of disaggregation into functional modules, as evidenced by BN-PAGE.

The *in silico* predicted structure suggests interactions between HoxH and HoxY subunits, forming a minimal hydrogenase module (HoxHY), as well as between HoxE, HoxU, and HoxF subunits, consistent with previous studies indicating oligomerization of the recombinant diaphorase module (HoxEUF) to a tetramer Hox(EUF)_4_ in the absence of HoxHY in *C. necator* HF903 (52). Additionally, HoxH and HoxY are attached to HoxU, allowing for complex formation and electron transfer between the minimal hydrogenase module HoxHY and the diaphorase module HoxUFE (Fig. 1*A*). Furthermore, the *in silico* model suggests that the HoxHYEUF heteropentamer dimerizes *via* HoxH, HoxU, and HoxE subunits, forming a mirrored and butterfly-shaped supercomplex, which aligns with the previously published structure of the soluble NAD^+^-dependent hydrogenase from *Hydrogenophilus thermoluteolus* TH-1 (PDB: 5XF9) (53). The HoxHYEUF pentamers are slightly twisted (∼30°) along the x-axis (Fig. 1*B*), while the N-terminus of HoxE is stretching towards HoxU, thus ‘embracing’ the opposite HoxHYEUF pentamer (Fig. 1*A*).

We positioned the metal cofactors and the FMN coenzyme by superimposing a calculated pentameric HoxHYEUF complex with the experimentally determined structure of the homologous soluble NAD^+^-dependent hydrogenase (PDB: 5XF9) of *H*. *thermoluteolus* TH-1, resulting in an RMSD of 1.48 Å. Identified conserved cystein residues were used to position the iron-sulfur clusters in the dimeric Hox(HYEUF)_2_ structure presented here. In addition, iron-sulfur clusters U4, E1, and F2 were predicted based on previous studies (52, 54) and were manually positioned in the *in silico* structure.

Nickel in HoxH is most probably coordinated by cystein residues 62, 65, 443, and 446. The proposed [4Fe-4S] cluster in HoxY (Y1) might be coordinated by Cys_14_, Cys_17_, Cys_87_, and Cys_151_. In Hox U, four iron-sulfur clusters are predicted: (*i*) U1, predicted as a [4Fe-4S] cluster (52), although only three conserved cystein residues (Cys_100_, Cys_103_, and Cys_109_) were found, suggesting a potential [3Fe-4S] cluster; (*ii*) U2, likely a [4Fe-4S] cluster coordinated by Cys_148_, Cys_151_, Cys_154_, and Cys_202_; (*iii*) U3, probably a [2Fe-2S] cluster coordinated by Cys_36_, Cys_47_, Cys_50_, and Cys_64_; and (*iv*) U4, likely a [4Fe-4S] coordinated by Cys_158_, Cys_192_, Cys_195_, and Cys_198_. In the HoxE subunit, one [2Fe-2S] cluster (E1) is predicted to be bound by Cys_96_, Cys_101_, Cys_137_, and Cys_141_. In the HoxF subunit, two iron-sulfur clusters are predicted: (*i*) F1, as a [4Fe-4S] cluster coordinated by Cys_458_, Cys_461_, Cys_464_, and Cys_504_; and (*ii*) F2, as a [2Fe-2S] cluster bound by Cys_25_, Cys_30_, Cys_62_, and Cys_66_.

The iron-sulfur clusters form an electrically conductive ‘wire’, with distances ranging from 9.8 Å (between U1 and U2) to 22.4 Å (between F1 and E1) (Fig. 1*B*). Fast electron transfer between metal redox centers is feasible for distances up to 14 Å (55). Thus, our *in silico* structure suggests that efficient electron transfer within the Hox complex is possible between most clusters, except between U3 and U4 (21.5 Å), F1 and E1 (22.4 Å), and between F1 or F2 and FMN (16.7 Å or 17.7 Å). Rapid electron transfer between these cofactors and coenzymes might be realized by conformational changes in the protein structure.

### Hox hydrogenase forms different active subcomplexes in the absence of oxygen

**Figure 2:**
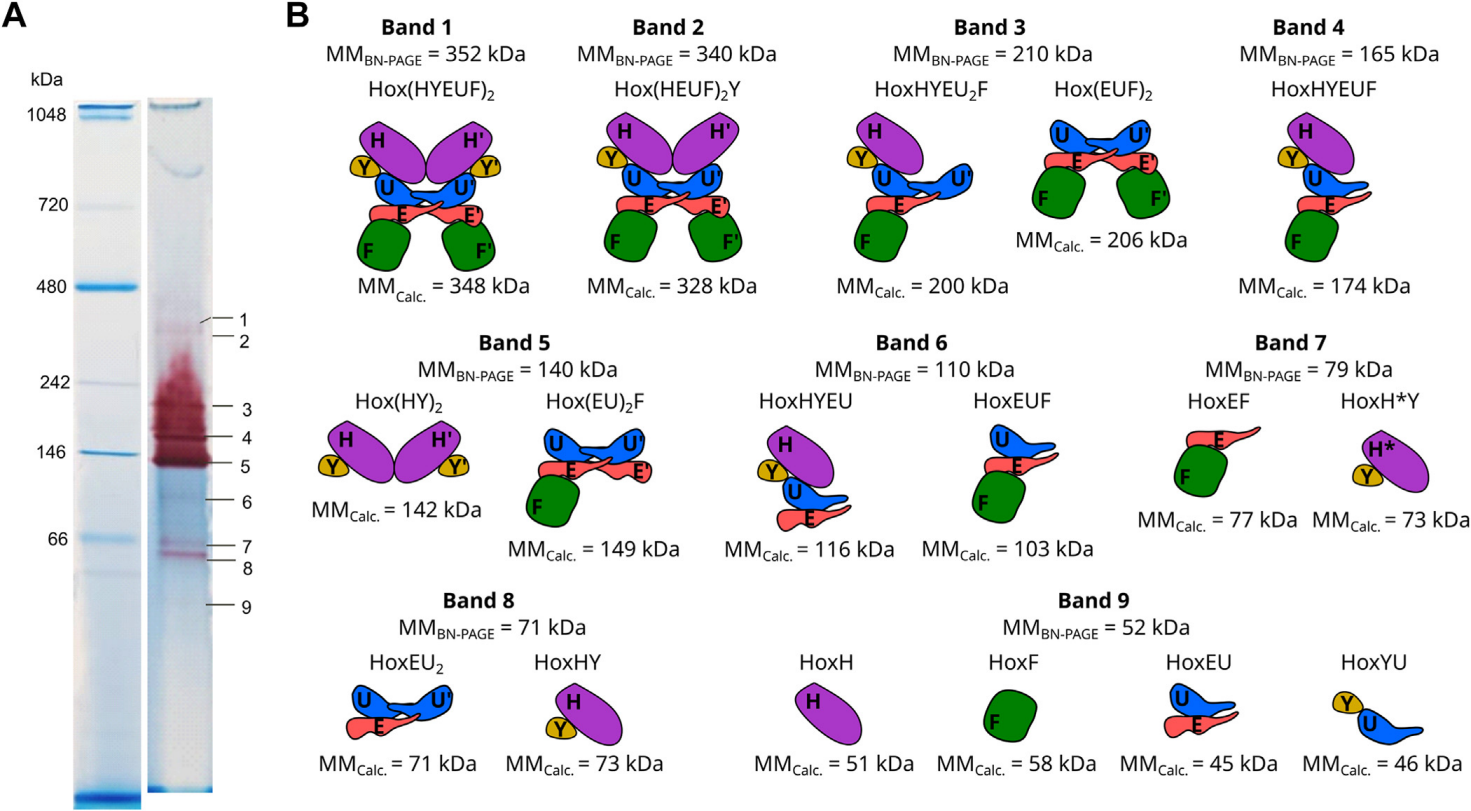
**Protein extracts from *Synechocystis* sp. PCC6803 separated by Blue Native PAGE, followed by in-gel hydrogenase activity staining and protein mass spectrometry (nLC-MS/MS).***A*, Hox hydrogenase in-gel activity staining using 1 mM benzyl viologen and 100% hydrogen. Active gel bands (1–9) were excised for nLC-MS/MS analysis. *B*, the schematic representation of various Hox hydrogenase subcomplexes, as observed through Blue Native PAGE. Molecular masses were determined using the Native Mark protein ladder (Invitrogen, MM_BN-PAGE_)_,_ and the calculated masses of individual subcomplexes (MM_Calc._) are provided. The formation of different subcomplexes (bands 1–9) is proposed based on dimerization involving HoxH (*purple*), HoxU (*blue*), and HoxE (*salmon*), as observed in the *in silico* predicted Hox(HYEUF)_2_ dodecameric complex. HoxY (*yellow*) is weakly attached to HoxH and HoxU, while HoxF (*green*) forms the ‘exit’ of the diaphorase module. HoxH represents the matured HoxH subunit, while HoxH∗ is the unprocessed subunit (band 7).

Electrons might be delivered to or accepted from the active site of Hox *via* different locations of the internal electron transport chain of the Hox complex (Fig. 1*B*). To identify active Hox subcomplexes in the crude extract of *Synechocystis* WT strain under anoxic conditions, proteins were separated by BN-PAGE, and Hox activity was assessed by in-gel activity staining in a 100% H_2_ atmosphere (electron donor) using BV as an electron acceptor (Fig. 2*A*). Active gel bands, distinguished by their red color, were excised, and proteins were identified by mass spectrometry, considering only those identified with a minimum of two unique peptides (Fig. S1). Subsequently, different Hox subcomplexes observed on the BN-PAGE were proposed using the *in silico* dodecameric Hox(HYEUF)_2_ complex structure predicted by AlphaFold 2 multimer (Fig. 1).

After in-gel activity staining, several faintly stained gel bands (1, 2, 6, 7, 8, 9) and three prominent gel bands (3, 4, 5) (Fig. 2*A*) were observed. Bands 1 and 2, with molecular masses (MM) of ∼352 kDa or ∼340 kDa, respectively, might comprise the intact Hox(HYUFE)_2_ dimer (348 kDa) or the Hox(HEUF)_2_Y subcomplex (328 kDa, bands 1–2 in Fig. 2*B*), although maximally three Hox subunits were identified *via* protein mass spectrometry, with MS1 intensities ranging from 3.1 × 10^5^ to 2.5 × 10^6^ (Fig. S1), indicating a very low protein content.

In bands 3 to 5, with MM of ∼210, ∼165, and ∼140 kDa, all five Hox hydrogenase subunits were identified *via* mass spectrometry, likely representing different active Hox subcomplexes. The ∼210 kDa band may contain the active HoxHYU_2_FE module (200 kDa) as well as an inactive Hox(EUF)_2_ submodule (206 kDa, band 3 in Fig. 2*B*), while the ∼165 kDa band may comprise the HoxHYUFE pentamer (174 kDa, band 4 in Fig. 2*B*). The ∼140 kDa band probably includes the active Hox subcomplex Hox(HY)_2_ (142 kDa), as well as the inactive subcomplex Hox(EU)_2_F (142 kDa, band 5 in Fig. 2*B*). In bands 3 to 5, HoxY and HoxE were detected only in low abundancies (Fig. S1), with MS1 intensities ranging from 3.4 × 10^5^ (HoxY in band 4) to 3.9 × 10^6^ (HoxY in band 5), whereas HoxH, HoxU, and HoxF subunits were detected more abundantly, with MS1 intensities between 6.6 × 10^6^ (HoxF in band 3) and 4.9 × 10^7^ (HoxU in band 5).

In protein band 6, with MM of ∼110 kDa exhibiting a very low in-gel Hox activity, four Hox subunits, excepting HoxY, were identified by mass spectrometry in low abundance (MS1 intensities ranging from 5.2 × 10^5^ for HoxE to 6.3 × 10^6^ for HoxU; Fig. S1), indicating that this protein band mostly comprises the inactive diaphorase module HoxEUF (103 kDa). Additionally, a small amount of HoxHYUE (116 kDa, band 6 in Fig. 2*B*) might be present, leading to weak in-gel activity staining.

Bands 7 and 8, with MM of ∼79 or ∼71 kDa, might contain the active HoxHY subcomplex, either in the unprocessed (73 kDa, band 7 in Fig. 2*B*) or matured (71 kDa) form (band 8 in Fig. 2*B*), as well as the inactive HoxEF and HoxEU_2_ subcomplexes (77 and 71 kDa, Fig. 2*B*). This is supported by the MS data, which identified HoxH, HoxU, and HoxF subunits in bands 7 and 8 in reasonable abundancies. However, HoxE was only detected in band 7 with a relatively low MS1 intensity of 6.1 × 10^5^ (Fig. S1).

In the low molecular weight gel band 9 of ∼52 kDa, all five Hox hydrogenase subunits were identified through mass spectrometry, indicating that band 9 most likely comprises the HoxH (51 kDa) and HoxF (58 kDa) monomers, as well as heterodimers HoxEU (45 kDa) and HoxYU (46 kDa, Fig. 2*B*). HoxH and HoxU were detected with reasonably high MS1 intensity of around 3 × 10^7^ (Fig. S1), although the in-gel Hox activity was very low, suggesting that the HoxH monomer is not highly active. However, the MS1 intensities of other subunits were much lower.

Although MS1 intensities of individual Hox subunits cannot provide absolute quantification, they serve as a direct measure of peptide abundance, allowing estimation of the relative amounts of proteins in each active band. BN-PAGE revealed that most Hox subunits, except HoxE, exhibited the highest MS1 intensities in protein band 5 (Fig. S1), indicating that Hox from *Synechocystis* was predominantly disaggregated into the active Hox(HY)_2_ heterodimer and other subcomplexes after BN-PAGE. The second highest MS1 intensities for Hox subunits were observed in protein bands 3 and 4, respectively, suggesting that a significant portion of the Hox complex was present as active HoxHYUFE pentamer (174 kDa, band 4 in Fig. 2*B*) or HoxHYU_2_FE submodule (200 kDa, band 3 in Fig. 2*B*). However, it remains unclear whether the disaggregation occurred during BN-PAGE or if these different Hox subcomplexes are naturally present in the cytosol of *Synechocystis*.

Taken together, in-gel hydrogenase staining displayed considerable intensity, particularly in bands 3 to 5 (Fig. 2*A*), the relative abundance of Hox subunits within all bands remained low when normalized to the intensities of other proteins detected in the individual gel bands. The relative abundances of all Hox subunits constituted no more than 0.5% in the most active protein bands 3 to 5 and up to 1.2% in other bands (Fig. S1). These findings suggest that while Hox hydrogenase expression levels were modest in *Synechocystis*, the enzyme exhibited high activity within the gel, particularly under a 100% H_2_ atmosphere.

### Hox hydrogenase disaggregates after oxygen exposure into different active subcomplexes

**Figure 3:**
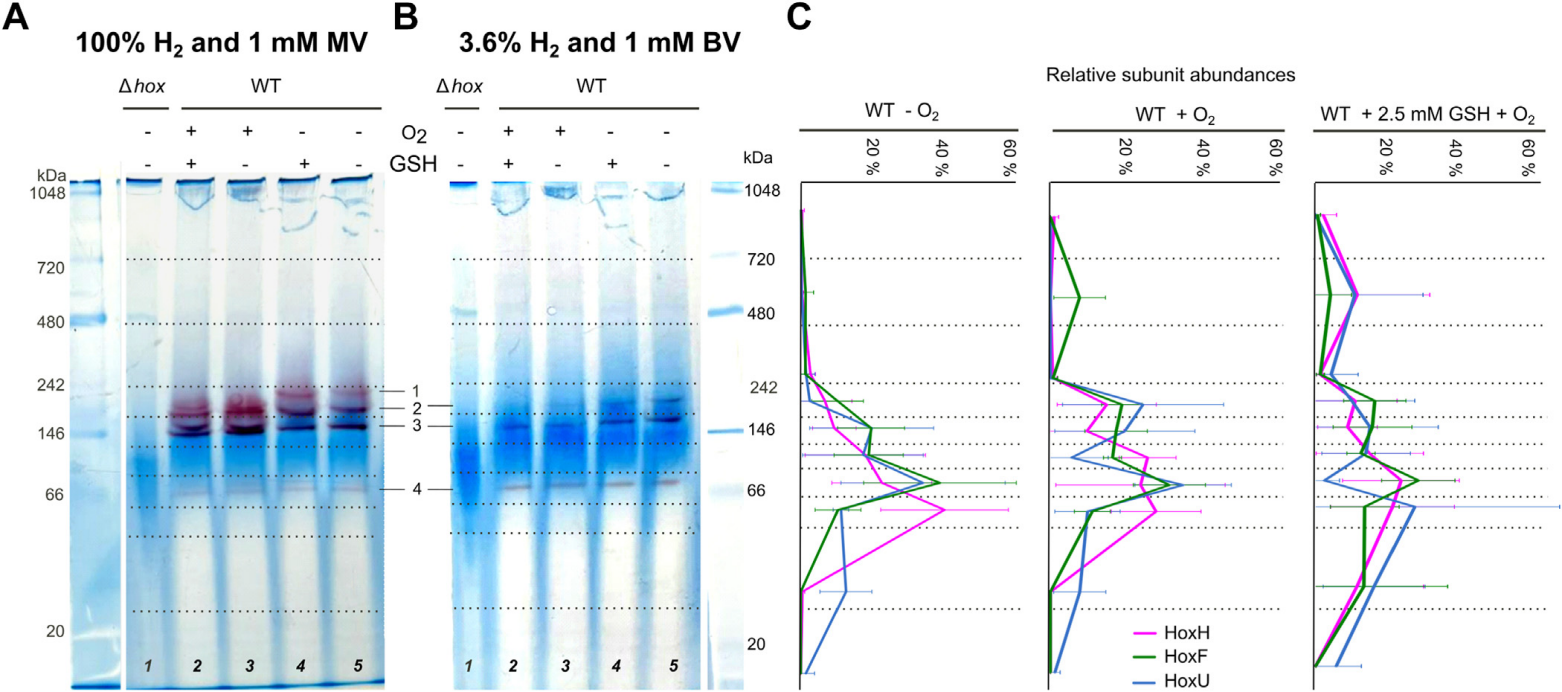
**Proteins from *Synechocystis* sp. PCC6803 WT and *hox*-deficient (Δ*hox*) strains separated *via* Blue Native-PAGE, followed by in-gel hydrogenase activity staining and complexome analysis.***Synechocystis’* protein crude extracts were exposed to oxygen (O_2_) agitating at 300 rpm for 30 min in ambient air and/or supplemented with 2.5 mM glutathione (GSH), as indicated by the + symbol. *A*, in-gel activity staining was conducted in the presence of 1 mM methyl viologen (MV), and 100% hydrogen (H_2_), or *B*, 1 mM benzyl viologen (BV) and 3.6% H_2_. *C*, the BN gel lane was cut into 10 slices (*black dotted* lines in (*A*) and (*B*)), and the relative abundancies (%) of HoxH, HoxU, and HoxF subunits in each gel slice were calculated by normalizing the MS1 intensity of each subunit to the total abundance of the respective subunit detected in the whole gel lane. Error bars represent the SD of three replicates per condition.

To assess the activity of Hox subcomplexes using methyl viologen (MV) as the electron acceptor under a 100% H_2_ atmosphere, we conducted in-gel activity staining, substituting MV for BV. The three most active Hox subcomplexes observed in Figure 2*A*, (bands 3–5) were also present when MV was used (Fig. 3*A*, lanes 4–5, bands 1–3). We then investigated whether these subcomplexes retain activity with H_2_ contents below 5%, which represents the H_2_ content in the anaerobic chamber expected during photometric assays. To identify the Hox subcomplexes that remain active under these conditions, in-gel activity staining utilizing 3.6% H_2_ and BV were conducted (Fig. 3*B*, lanes 4–5). Under these conditions, the ∼210 kDa band disappeared (Fig. 3*B*, band 1), indicating that the putative HoxHYU_2_FE subcomplex (200 kDa, Fig. S1*C*) may require high H_2_ partial pressure to exhibit *in vitro* activity. However, bands at ∼165 kDa and ∼140 kDa (Fig. 2, bands 2 and 3), putatively comprising the HoxHYUFE pentamer (174 kDa, Fig. S1*D*) and the active minimal Hox subcomplex Hox(HY)_2_ (142 kDa, Fig. S1*E*), as well as the band around ∼71 kDa (Fig. 2, band 4), probably containing the matured HoxHY (71 kDa, Fig. S1*G*), exhibited activity with 3.6% H_2_ (Fig. 3*B*). The experiments using MV as electron acceptor in the presence of 100% H_2_ respectively were repeated multiple times, consistently resulting in the formation of lower molecular weight subcomplexes in oxygen exposed samples.

Subsequently, the effects of oxygen and GSH exposure on the in-gel hydrogenase activity and structural integrity of the Hox complex in both the WT and Δ*hox* mutant strains of *Synechocystis* were investigated. No activity-stained bands were observed in crude extracts from the Δ*hox* mutant strain deficient in *hox* genes (Fig. 3, *A* and *B*, lane 1), demonstrating the specificity of both viologen-based in-gel staining approaches for Hox.

In the MV-stained gel using 100% H_2_, the active band at ∼210 kDa (Fig. 3*A*, band 1) vanished when samples were exposed to oxygen before BN-PAGE (Fig. 3, *A* and *B*, lanes 2–3). New bands, directly above the ∼165 and ∼140 kDa bands (Fig. 3*A*, bands 2 and 3), appeared forming a double band, which indicates potential disaggregation of the HoxHYU_2_FE subcomplex (200 kDa, Fig. S1*C*) into smaller active Hox subcomplexes after oxygen exposure. In the BV-stained BN gel using 3.6% H_2_, the ∼165 kDa band (Fig. 3*B*, band 2) corresponding to the HoxHYUFE pentamer (174 kDa, Fig. S1*D*) disappeared after oxygen exposure (Fig. 3*B*, lane 2–3). The activity in this band could not be regenerated by 3.6% H_2_ or by GSH supplementation (Fig. 3*B*, lane 2–3). In contrast, the activity of this subcomplex was restored in the presence of 100% H_2_ during the staining procedure (Fig. 3*A*, lane 3, band 2). However, GSH addition in oxygen-exposed samples did not alter the staining pattern, neither in the MV-stained BN gel incubated with 100% H_2_ nor in BV-stained gel incubated with 3.6% H_2_ (Fig. 3, *A* and *B*, lane 2), respectively. Nevertheless, in-gel Hox activity staining indicated that the H_2_-driven MV or BV reduction activity of selected Hox subcomplexes was restored, as activity-stained bands persisted after exposure to oxygen (Fig. 3, *A* and *B*).

After BN-PAGE and in-gel Hox activity staining, the gels were sliced into 10 pieces, and complexome analysis was conducted by mass spectrometry (Fig. 3*C*). However, complexome analysis revealed no significant differences in subunit distribution under different conditions (Fig. 3*C*). The three subunits HoxH, HoxF, and HoxU were consistently identified under all conditions across a wide range of molecular weights, from 240 to 50 kDa, including the activity-stained region. Conversely, the HoxY and HoxE subunits were either not identified or only found in one replicate due to very low protein abundances, as previously demonstrated for the sliced protein bands (Fig. 2*B*).

However, the catalytic subunit HoxH was predominant in the gel band at ∼50 kDa (Fig. 3*C*), indicating significant disaggregation of the Hox complex. This finding is supported by mass spectrometric data obtained from BV-stained gel bands, where relatively high amounts of all five Hox subunits were identified in bands excised from the low molecular weight region of the gel (Fig. 2*B*). However, our current data from in-gel activity staining and complexome analysis suggest that we cannot entirely exclude the possibility that Hox complex disaggregation could be influenced by factors such as the Coomassie additive used during BN-PAGE or whether all these different subcomplexes naturally occur in the cytosol of *Synechocystis*.

### Glutathione impacts hox activity under both oxygen-exposed and nonexposed conditions

**Figure 4:**
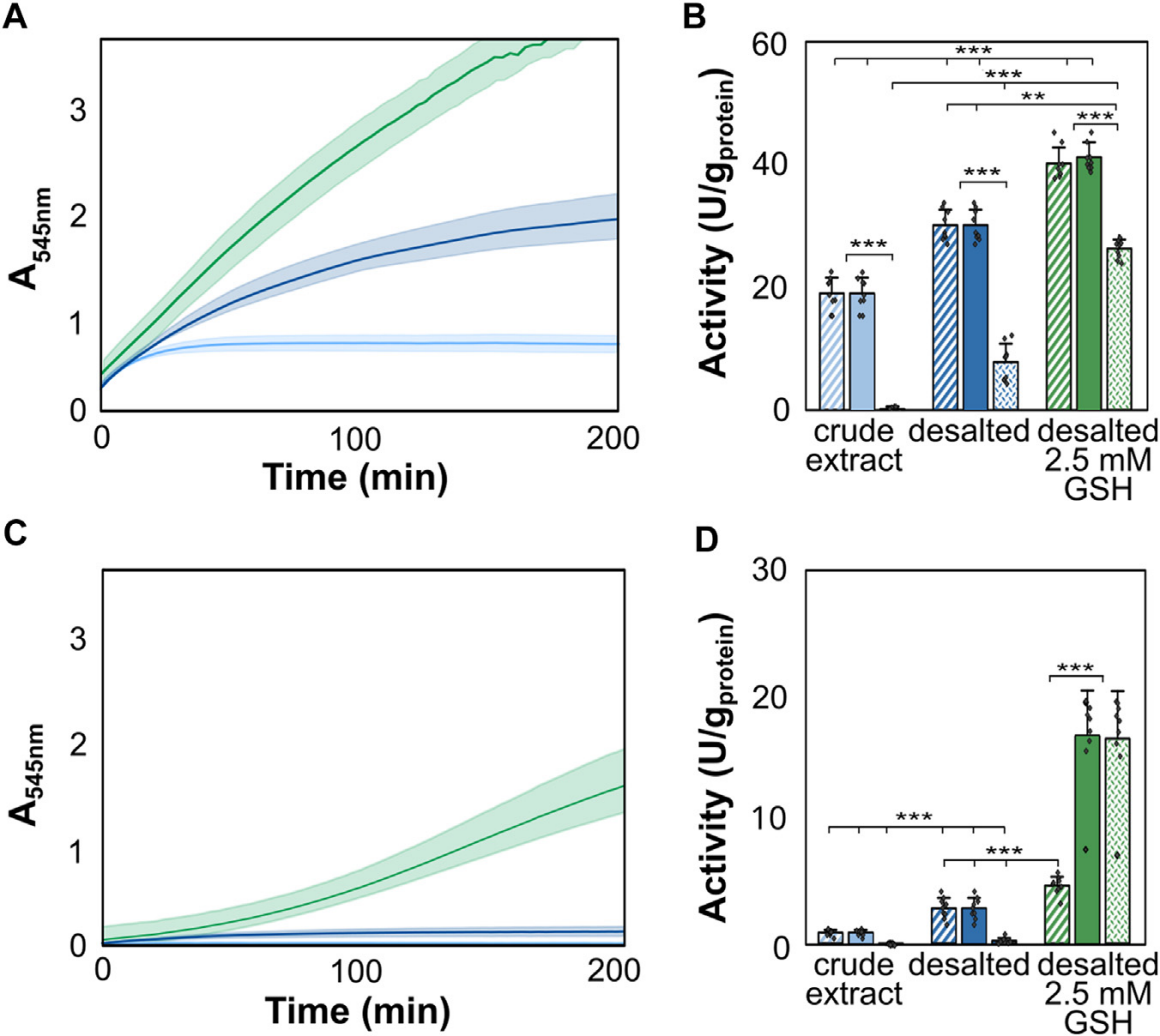
**Photometric Hox activity assays in crude extracts from *Synechocystis* sp. PCC6803 measured by the increase in absorbance at 545 nm (A**_**545nm**_**).** Nondesalted protein crude extracts are represented in *light blue*, desalted crude extracts without glutathione (GSH) or with 2.5 mM GSH are shown in *dark blue* or *green*, respectively. Initial activity (dashed columns) was calculated within the first 30 min of incubation, the maximal activity (filled columns) is the highest activity throughout any 30-min time interval, and the long-term activity (dotted columns) was calculated between 120 and 150 min of incubation. *A*, absorbance changes at 545 nm for samples not exposed to oxygen. The shaded area encompasses the SDs, and the average of nine replicates (n = 9) is shown as a line. *B*, the initial, maximal, and long-term Hox activities for samples not exposed to oxygen. *C*, the increase of absorbance at 545 nm in samples exposed to air for 3 to 5 s. *D*, the initial, maximal, and long-term Hox activity of oxygen-exposed samples. Significances were determined using the unpaired Student’s *t* test with ∗∗: *p* < 0.01 and ∗∗∗: *p* < 0.001.

In-gel Hox activity staining represents an endpoint activity measurement, from which the dynamic impact of oxygen and/or GSH on enzyme kinetics cannot be inferred. To investigate the dynamic impact of GSH on Hox activity and kinetics, we conducted photometric enzyme activity assays, determining H_2_-driven BV reduction rates at 545 nm in crude extracts from *Synechocystis*. These measurements enabled the calculation of different specific enzyme turnover rates (activities), normalized to protein content: (*i*) initial activity of Hox, reflecting the enzyme’s activity within the first 30 min of incubation, (*ii*) maximal Hox activity, determined as the highest activity measured within any 30-min time window throughout the entire measurement, and (*iii*) long-term Hox activity, reflecting the enzyme’s activity between 120 and 150 min of incubation (Table S1). Subsequently, we compared the specific activities between samples exposed and unexposed to oxygen, with and without the addition of GSH, alongside specific activities observed in nondesalted extracts, mirroring the cytosolic conditions in *Synechocystis* (Fig. 4). Based on intracellular concentrations of GSH reported by Cameron *et al.* (26), we chose a concentration of 2.5 mM GSH in activity assays.

Specific Hox activities were determined in cell-free protein preparations (crude extracts), desalted with HiTRAP desalting column (Cytiva) for the removal of reducing agents and other low molecular weight compounds. In desalted samples not exposed to air and supplemented with 2.5 mM GSH, a continuous absorbance increase at 545 nm was observed, indicating sustained Hox activity (Fig. 4*A*, green line), consistent with previous study (56). In contrast, the activity in desalted samples without GSH diminished after around 100 min (Fig. 4*A*, dark blue line), while the activity ceased after approximately 30 min in nondesalted samples (Fig. 4*A*, light blue line). Consequently, desalting the crude extracts in anoxic buffer significantly increased the maximal Hox activity by approximately 60%, from 19 ± 2 U/g_protein_ to 30 ± 2 U/g_protein_, and the long-term activity by approximately 78%, from 0.1 ± 0.4 U/g_protein_ to 8 ± 3 U/g_protein_. The positive effect of desalting on Hox activity might be due to the removal of inhibitory components from the crude extract and the exchange to an anoxic buffer that might be potentially more favorable for Hox activity than the cytosolic environment of *Synechocystis*. Further supplementation of desalted extracts with 2.5 mM GSH enhanced the maximal and long-term activity significantly, by around 30% and 70%, respectively, resulting in 41 ± 2 U/g_protein_ and 26 ± 1 U/g_protein_. Thus, GSH addition to desalted samples improved both the maximal activity and long-term hydrogenase activity (Fig. 4, Table S1) in samples not exposed to oxygen.

Given that Hox exhibits long-term deactivation when exposed to oxygen for 3 to 5 s only and that reactivation post oxygen exposure is facilitated by low redox potentials (43), we investigated the impact of GSH on Hox activity in samples exposed to air for 3 to 5 s. Compared to anoxic samples, significant inhibition of Hox activity was observed in both desalted and untreated crude extracts (Fig. 4*C*, light and dark blue lines). Initial activities in desalted samples without GSH were 3 ± 1 U/g_protein_ and 5 ± 1 U/g_protein_ in samples with GSH, corresponding to similar activity recoveries of 10% and 13%, respectively. Interestingly, although in-gel activity staining did not reveal any changes in the staining pattern for samples exposed to oxygen and treated with GSH (Fig. 3*A*, lane 2), a notable recovery in Hox activity after approximately 120 min of incubation was observed in photometric assays (Fig. 4, *C* and *D*, green line). This resulted in maximal and long-term activities of around 17 ± 4 U/g_protein_ (Fig. 4*D*), representing approximately 40% and 65% maximal and long-term activity recoveries compared to GSH-supplemented samples not exposed to oxygen (Fig. 4*B*; Table S1).

To ensure that the observed BV reduction kinetics were not influenced by other proteins in the *Synechocystis* crude extracts or by GSH addition, various controls were implemented: (*i*) a no-enzyme-control (NEC) containing buffer instead of protein extract, with or without GSH, showed no absorbance changes at 545 nm; (*ii*) H_2_-dependent BV reduction background activity not related to Hox was assessed using Δ*hox* strain extracts, showing no changes in absorbance at 545 nm (data not shown); (*iii*) BV re-oxidation background activity not related to Hox hydrogenase was determined using Δ*hox* crude extracts (Fig. S3*A*); and (*iv*) the stability of reduced BV in the presence and absence of GSH was monitored (Fig. S3*B*). For controls (*iii*) and (*iv*), 0.1 mM BV was reduced with titanium(III)citrate and absorbance changes at 545 nm were tracked over 200 min. While Δ*hox* crude extract caused a decrease in absorbance, indicating BV oxidation through enzymes not related to Hox (Fig. S3*A*), the absorbance remained stable in protein-free buffer with or without GSH (Fig. S3*B*).

**Figure 5:**
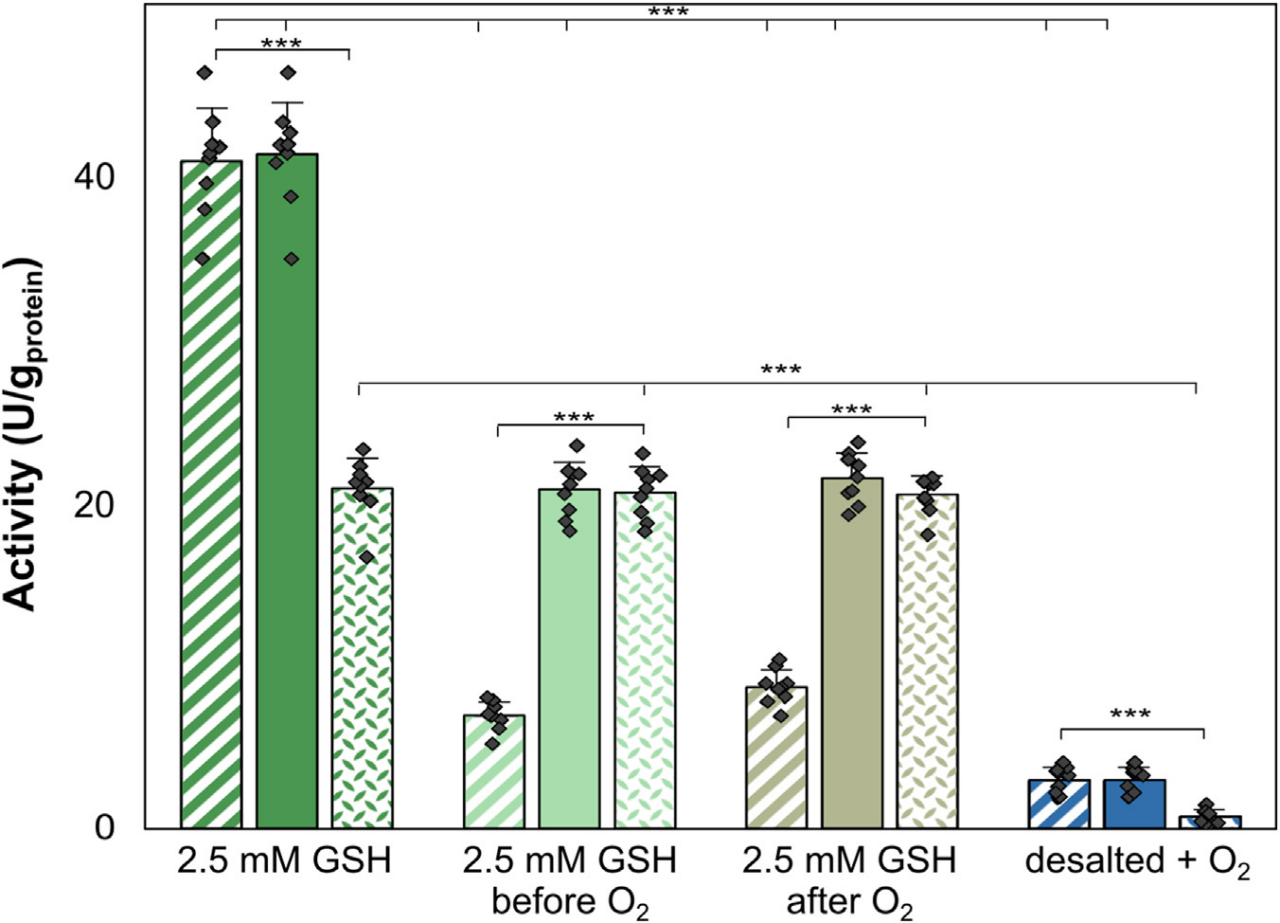
**The influence of glutathione (GSH) on Hox activity in the crude extracts of *Synechocystis* sp. PCC6803 with GSH addition either before or after exposure to oxygen (O**_**2**_**).** Protein crude extracts were desalted, and Hox activity was assessed photometrically using benzyl viologen (BV) as electron acceptor by monitoring absorbance changes at 545 nm. Initial activity (dashed columns), calculated within the initial 30 min of incubation, the maximal activity (filled columns), calculated as the highest activity throughout any 30-min time window, and the long-term activity (dotted columns), calculated between 120 and 150 min of incubation, are compared. Hox activity of desalted samples with 2.5 mM GSH before exposure to O_2_ (*light green*) was compared with Hox activities in crude extracts where GSH was added after O_2_ exposure and re-establishment of anoxic conditions (olive). The activity of desalted GSH-free samples exposed to O_2_ is shown in *blue*, while the activity of samples amended with GSH and not exposed to O_2_ is in *dark green*. The average of nine replicates and SDs are shown. Significances were determined using the unpaired Student’s *t* test with ∗∗∗: *p* < 0.001.

To investigate if the effect of GSH on Hox activity depends on its addition before or after oxygen exposure, two sample sets were analyzed. One set was supplemented with 2.5 mM GSH prior to a 3 to 5 s exposure to oxygen, while the other set was exposed to oxygen first and then amended with 2.5 mM GSH. Both sets exhibited comparable maximal Hox activities of approximately 21 ± 1 U/mg_protein_, recovering after about 120 min of incubation (Fig. S4*A*), with significantly lower initial activities at the onset of incubation (7–9 U/mg_protein_). Consistent with previous experiments (Fig. 4), adding 2.5 mM GSH, whether before or after oxygen exposure, resulted in about 50% recovery of maximal activity and 100% recovery of long-term activity compared to GSH-supplemented samples not exposed to oxygen, which showed about 41 ± 2 U/mg_protein_ maximal and 21 ± 2 U/mg_protein_ long-term Hox activities (Fig. 5, Table S1).

We repeated the experiments multiple times and consistently observed a protective effect of GSH in comparison to non-GSH–supplemented samples. This included reactivation, improved recovery of maximal Hox activity, and stabilization of long-term Hox activity. However, the extent of these protective effects varied among different cell preparations, highlighting the importance of analyzing the multifactorial complexity of the system.

### Different glutathione concentrations have an impact on Hox activity after oxygen exposure

**Figure 6:**
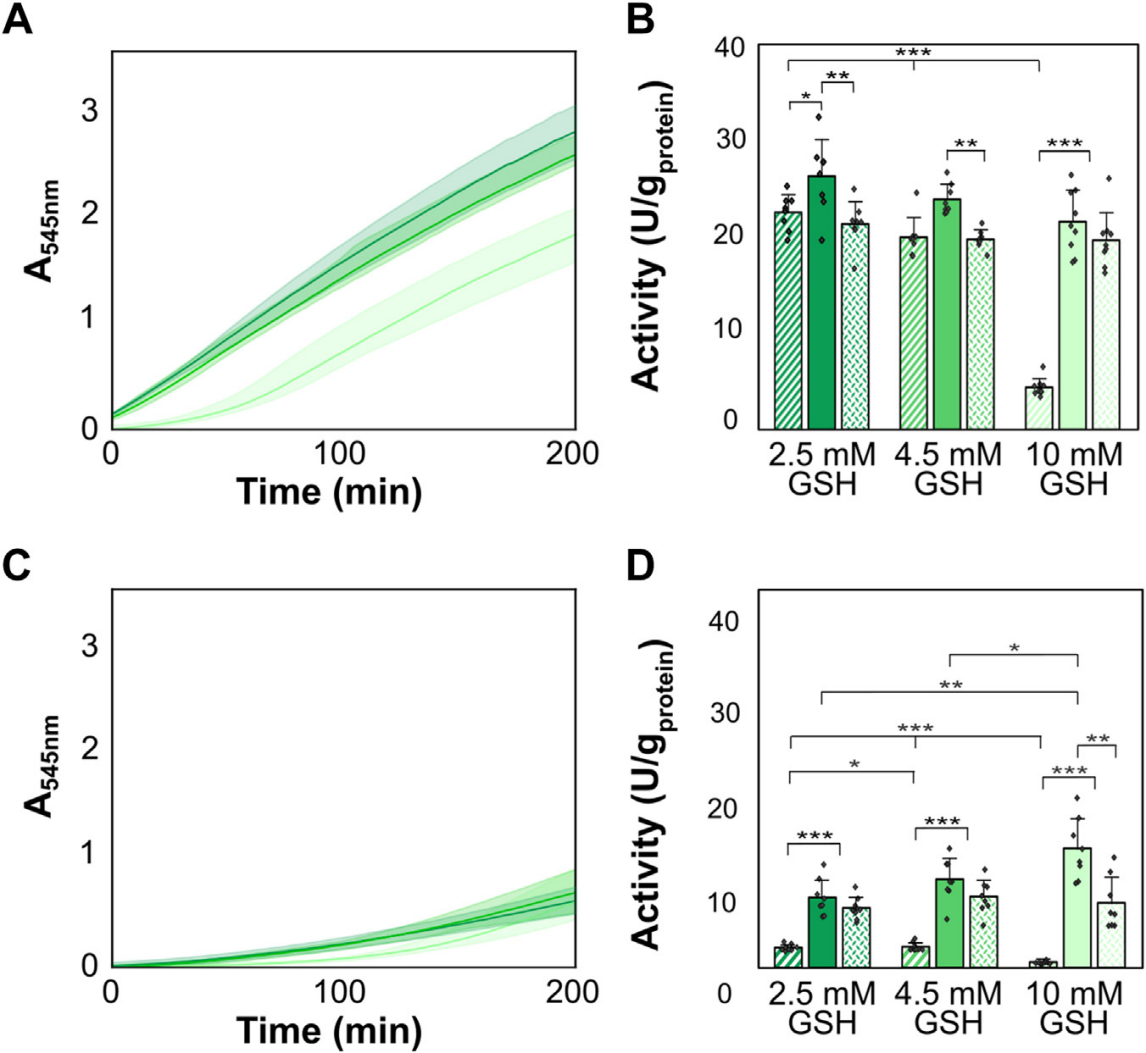
**Impact of varying glutathione (GSH) concentrations on Hox activity in desalted crude extracts of *Synechocystis* sp. PCC6803, measured as absorbance changes at 545 nm (A**_**545nm**_**).** Desalted crude extracts with 2.5 mM GSH are depicted in *dark green*, 4.5 mM GSH in *medium green*, and 10 mM GSH in *light green*. Activities in the initial 30 min of the assay (initial activity, *dashed* columns), in the time window between 120 and 150 min (long-term activity, *dotted* columns) were calculated alongside the maximal activity (*filled* columns), defined as the highest activity throughout any 30-min time window. *A*, the increase in absorbance at 545 nm resulting from benzyl viologen (BV) reduction for samples not exposed to oxygen, and *C*, for samples exposed to oxygen for 3 to 5 s. The shaded area represents SDs, with the average of eight replicates (n = 8) shown as a line. *B* and *D*, maximal hydrogenase activities calculated as highest activities in any possible 30-min time interval during the entire measurement period. Significances were determined using the unpaired Student's *t* test for normally distributed data sets and the Mann-Whitney U-test for non-normally distributed samples, with ∗: *p* < 0.05, ∗∗: *p* < 0.01, and ∗∗∗: *p* < 0.001.

As intracellular GSH concentrations fluctuate, we investigated the impact of different GSH concentrations and oxidized glutathione (GSSG) on Hox activity *in vitro*. In desalted samples not exposed to oxygen, elevated GSH concentration led to a prolonged *lag* phase for the initiation of H_2_-driven BV reduction activity (Fig. 6*A*). Particularly, in samples amended with 10 mM GSH and not exposed to oxygen, the maximal activity decreased significantly by around 15% compared to those treated with 2.5 mM GSH (22 ± 3 U/mg_protein_ *vs.* 26 ± 4 U/mg_protein_) (Fig. 6*B*).

When desalted protein crude extracts from *Synechocystis* were exposed to oxygen, an opposite effect was observed: higher GSH concentrations resulted in steeper slopes in absorbance changes at 545 nm (Fig. 6*C*), leading to increased maximal Hox activity (Fig. 6*D*). The addition of 10 mM GSH led to a significantly higher maximal activity recovery of around 61% than 29% with 2.5 mM GSH in oxygen-exposed samples (Fig. 6*D*, Table S1). However, increasing GSH concentrations beyond 2.5 mM did not further enhance long-term activity, which remained stable between 6 ± 3 U/mg_protein_ (with 10 mM GSH) and 6 ± 1 U/mg_protein_ (with 2.5 mM GSH), resulting in an approximate long-term activity recovery of 26% (Table S1).

**Figure 7:**
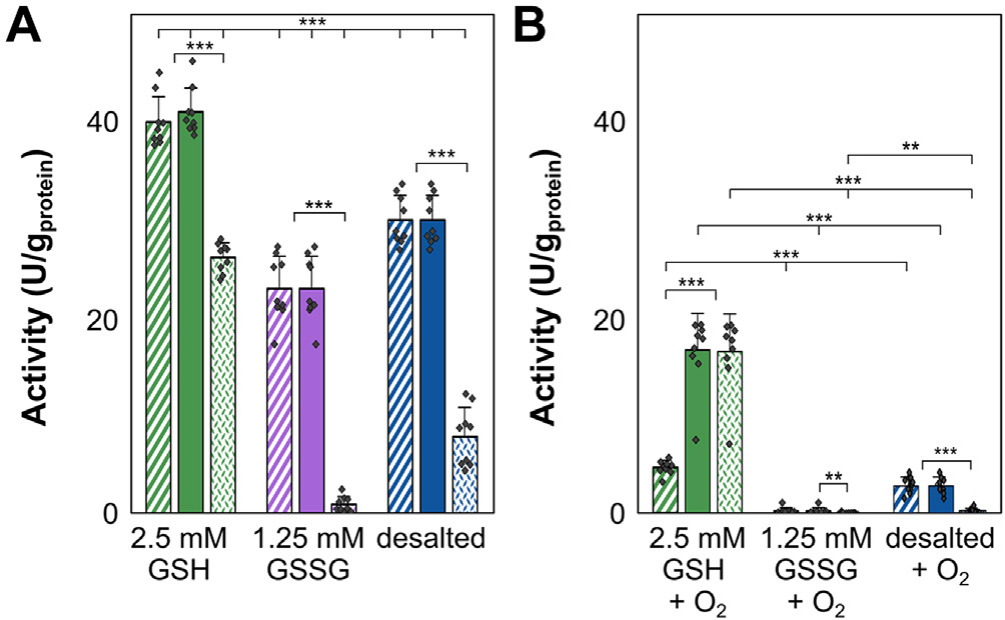
**The impact of reduced (GSH) and oxidized (GSSG) glutathione on Hox activity in desalted crude extracts of *Synechocystis* sp. PCC6803, measured by absorbance changes at 545 nm.** Samples supplemented with 2.5 mM GSH are in *green*, samples with 1.25 mM GSSG in *violet*, and desalted samples without additives in *blue*. Initial activity (*dashed* columns) was calculated within the first 30 min of incubation, the maximal activity (*filled* columns) is the highest activity throughout any 30-min time interval, and the long-term activity (*dotted* columns) was calculated between 120 and 150 min of incubation. *A*, initial, maximal, and long-term Hox activities from nine replicates per condition not exposed to oxygen. *B*, initial, maximal, and long-term Hox activities of crude extracts exposed to air for 3 to 5 s from nine replicates per condition. Significances were determined using the unpaired Student's *t* test for normally distributed data sets and the Mann-Whitney U-test for non-normally distributed samples, with ∗∗: *p* < 0.01 and ∗∗∗: *p* < 0.001.

In contrast to the beneficial impact of GSH, the addition of oxidized glutathione (GSSG) to desalted crude protein extracts not exposed to oxygen significantly reduced the initial and maximal Hox activities by around 24%, resulting in 23 ± 3 U/mg_protein_ compared to 30 ± 2 U/mg_protein_ in desalted, GSH/GSSG-free samples (Fig. 7*A*, Table S1). Additionally, the presence of GSSG caused a rapid decrease in long-term activity by around 96% to 1 ± 1 U/mg_protein_, compared to 26 ± 1 U/mg_protein_ with reduced GSH (Fig. S4, *C* and *D*, Table S1). Moreover, GSSG had a detrimental effect on Hox activity in oxygen-exposed extracts, resulting in a complete loss of initial, maximal, and long-term activities. In contrast, desalted samples with GSH maintained maximal and long-term activities of around 17 ± 1 and 16 ± 4 U/mg_protein_, respectively (Fig. 7*B*, Table S1). This is also mirrored in a loss of residual activity, calculated from the maximal activity after oxygen exposure and normalized by the anoxic activity under the same conditions. While residual activity was 41% in the presence of GSH, it was 1% for samples amended with GSSG (Fig. S5*A*). Desalted samples without GSH also showed very low maximal and long-term activities of 3 ± 1 and 0.2 ± 0.2 U/mg_protein_, respectively (Table S1).

Our data highlight the distinct and contrasting effects of GSH and GSSG on Hox activity, underscoring the crucial role of redox status in modulating the enzyme’s functionality under both anoxic and oxygenic conditions.

### Thiol-containing reducing agents impact Hox activity after oxygen exposure

**Figure 8:**
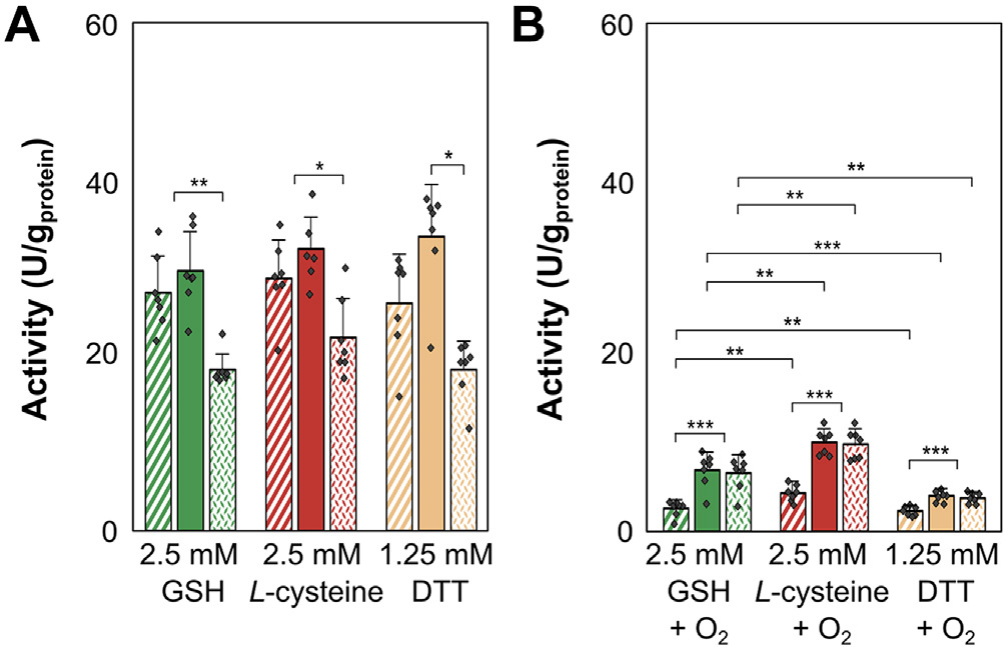
**Hox activity in desalted protein crude extract of *Synechocystis* sp. PCC6803 in the presence of 2.5 mM glutathione (GSH) (*green*), 2.5 mM *L*-cysteine (*red*), and 1.25 mM DTT (*yellow*).** The initial activities, shown as *dashed* columns, were calculated in the first 30 min of measurement. Maximal activity (*filled* columns) was determined as the highest activity within any 30-min interval, while the long-term activity (*dotted* columns) was assessed between 120 and 150 min of incubation. *A*, Hox activities from seven replicates (n = 7) each condition not exposed to oxygen. *B*, Hox activities measured after short exposure to oxygen for 3 to 5 s. Statistical significances were determined using the unpaired Student's *t* test for normally distributed data sets and Mann-Whitney u-test for non-normally distributed samples with ∗: *p* < 0.05, ∗∗: *p* < 0.01, and ∗∗∗: *p* < 0.001.

To determine whether the observed positive effects on Hox activity from *Synechocystis* are specific for GSH, we compared different thiol-containing reducing agents for their potential to regenerate Hox activity after oxygen exposure (Fig. 8). In addition to 2.5 mM GSH as a positive control, we used 2.5 mM *L*-cysteine (*L*-Cys) and 1.25 mM DTT, with the DTT concentration halved because it has two free thiol groups, while other agents have only one.

Initial and maximal Hox activities in samples not exposed to oxygen did not show significant differences between those amended with GSH, *L*-Cys, or DTT (Fig. 8*A*, Table S1). Furthermore, long-term Hox activities in these samples (not exposed to oxygen) were also comparable regardless of the additive used (Fig. S4, *A* and *B*, Table S1).

All thiol-containing reducing agents tested in our study positively affected Hox activity from *Synechocystis* after oxygen exposure, enhancing both maximal and long-term activities (Fig. 8*A*). Maximal Hox activity in oxygen-exposed samples recovered to around 23% with GSH, 32% in *L*-Cys-supplemented samples, and 13% with DTT compared to non-oxygen–exposed samples with the same reducing agents (Figs. 8*B*, S5*B*, Table S1). The addition of these agents also significantly stabilized long-term Hox activities, with recovery rates ranging from 21% (with 1.25 mM DTT) to 45% (with 2.5 mM *L*-Cys) compared to non-oxygen–exposed extracts containing the same reducing agents (Fig. 8*B*, Table S1). The most notable result of our experiments is the tremendous recovery of long-term Hox activity due to the addition of thiol-containing reducing agents compared to oxygen-exposed samples without these agents. While desalted, oxygen-exposed samples without thiol-containing reducing agents exhibited almost no long-term Hox activity (0.2 ± 0.2 U/mg_protein_) (Figs. 5, 7*B*, and Table S1), samples treated with 2.5 mM GSH, 1.25 mM DTT, and 2.5 mM *L*-Cys demonstrated significantly higher long-term activities, measuring 7 ± 2 U/mg_protein_ (with GSH), 4 ± 1 U/mg_protein_ (with DTT), or 10 ± 2 U/mg_protein_ (with *L*-Cys), respectively.

## Discussion

The potential for biotechnological H_2_ production coupled with photosynthesis has led to intensive studies of hydrogenases in photosynthetic microorganisms. These enzymes are investigated either in whole cells (37, 57), measuring H_2_ oxidation or formation, or through the use of purified enzymes (43, 58).

Whole-cell or crude extract studies using native electron donors or acceptors resemble a black box model, where observed hydrogenase activities result from various simultaneous cellular mechanisms, making data interpretation challenging (26, 37, 43). Specifically, varying autotrophic/mixotrophic growth conditions or the exposure of *Synechocystis* cells or crude extracts to chemicals and redox agents can significantly alter the intracellular environment, likely affecting Hox activity and (sub)complex formation and may add artefacts from mass transfer (uptake) complicating data analysis. Measuring *in vivo* activities in intact cells or crude extracts exposed to fluctuating oxygenic environments introduces variability that is difficult to control, hindering reliable conclusions. To address this, we used cell-free crude extracts from standardized culturing conditions, thereby minimizing variations in Hox integrity from cultivation. Our methodology, including removal of low-molecular-weight agents, ensured that we obtained Hox-containing protein fractions of consistent quality, allowing us to effectively study the effects of GSH and GSSG on Hox activity and complex integrity. Additionally, using *in vitro* H_2_-based BV reduction assays with photometric detection, instead of *in vivo* assays, enabled precise monitoring of activity kinetics, capturing distinct reaction phases such as inactivation, reactivation, and both maximal and long-term activity. Another advantage of the photometric approach is that it enables us to specifically track Hox hydrogenase activity in *Synechocystis* crude extracts, avoiding background interference from other enzyme activities (*e.g.*, NADH-dependent enzymes). Since the native electron donors and acceptors for Hox hydrogenase *in vivo* remain inconclusive due to inconsistent data, the photometric approach using H_2_ as the electron donor and BV as the electron acceptor offers clear advantages over more laborious and time-consuming *in vivo* assays.

In contrast, studies with purified enzymes allow precise characterization of isolated parameters, mostly on nonsystem levels. In addition, results derived from purified hydrogenases are challenging and may be biased by artefacts due to protein complex disaggregation, as has been also observed in our study (Fig. 3). PFE has gained popularity in characterizing hydrogenases (33, 59). In PFE, purified enzymes are immobilized on an electrode, and their reaction mechanism, deactivation and reactivation rates, turnover rates, and redox potentials are determined by current flow. Despite its value, PFE is influenced by factors such as the efficiency of interfacial electron transfer between the enzyme and the electrode, heterogeneous protein distribution and orientation on the electrode, and the unnatural local environment of the electrode, which differs from natural cellular conditions. Overlapping signals from different protein subcomplexes or redox-active sites can further complicate PFE analysis (60). Additionally, embedding the enzyme within an electrode matrix may shield enzyme regions from the environment, potentially affecting studies on the effects of oxygen or other additives on hydrogenase activity under physiological conditions (59). Our study aimed to analyze the activities of Hox from *Synechocystis* in its natural reaction environment, focusing on the redox effect of GSH, instead of an artificial electrode effect in PFE. We used GSH concentrations mimicking intracellular concentrations for understanding the reactivation of Hox after oxygen exposure under conditions closely resembling its physiological environment. The developed workflow involving BN-PAGE followed by Hox in-gel activity staining and photometric assays allowed the determination of dynamic H_2_ oxidation activities in protein crude extracts of *Synechocystis* without possible artefacts derived from purified Hox hydrogenase. Using viologen-based *in vitro* Hox activity assays, we demonstrated that GSH modulates the activity of the oxygen-sensitive bidirectional [NiFe] Hox hydrogenase of *Synechocystis*, enhancing maximal Hox activity and stabilizing long-term activities (Figs. 4 and 5, Table S1). Notably, after oxygen exposure, GSH reactivated and enhanced Hox activity in a concentration-dependent manner (Fig. 5). Alternative thiol reducing agents, *L*-Cys and DTT, showed similar beneficial effects (Fig. 7), whereas GSSG adversely affected Hox activity and reduced oxygen tolerance of Hox (Fig. 6). Although the beneficial effect of GSH on H_2_ oxidation may not directly translate to H_2_ production due to differing redox optima (43), the recovery of Hox activity after oxygen exposure is likely to be transferable to biotechnological applications.

Most hydrogenases are either partially or completely inactivated by oxygen. Two types of oxygen-induced deactivation have been identified: (*i*) oxygenation, where oxygen attaches to the enzyme without reacting and allowing reactivation by simple oxygen removal (61) and (*ii*) oxidation, where oxygen chemically reacts with the enzyme, forming, *for example*, a hydroxy-bridge with the nickel and iron ions in the active site (61, 62). Reactivation after oxygenation might occur rapidly by removing oxygen, but inhibition caused by oxidation requires longer reactivation. Reduction of enzymatic activity in hydrogenases after oxygen exposure likely involves a combination of both effects. In our study, both effects were observed, with the inhibitory oxygenation effect likely seen in nondesalted crude extracts exhibiting significantly lower initial, maximal, and long-term Hox activities compared to desalted samples (Fig. 4, Table S1). Desalting in anoxic buffer significantly increased these activities, possibly due to the removal or fivefold dilution of inhibitory components and the replacement of oxygen-containing cytosol with an anoxic buffer, thereby facilitating reactivation after oxygenation. Hox inactivation through oxidation was probably evident after 3 to 5 s of oxygen exposure, resulting in a substantial decrease in initial Hox activity, indicated by a *lag* phase of 30 to 100 min (Figs. 4 and 5). This indicates reactivation following oxidative damage rather than mere oxygenation. The addition of GSH to oxygen-exposed samples not only restored Hox activity, resulting in increased maximal activity, but also significantly stabilized long-term activity (Figs. 4*B* and 6, Table S1).

The protective effect of GSH, *L*-Cys, and DTT observed after oxygen exposure in our study could be attributed to various mechanisms. Reduced thiol groups of GSH, *L*-Cys, and DTT may act as oxygen scavengers, hindering oxygen from reaching the enzyme’s active site and reducing enzyme oxygenation, as described previously (56, 63). However, abiotic experiments did not show differences in oxygen content between buffers with and without GSH (data not shown), indicating that the protective effect may not be due to changes in oxygenation state. To fully exclude oxygenation effects, determining oxygen concentration within the enzyme’s active site in the presence or absence of GSH would be necessary, which is currently unfeasible.

GSH may protect Hox by altering the redox potential, as shown in prior research (43, 64). Our data align with these studies, indicating GSH’s positive impact on oxygen tolerance through redox potential reduction, contrasting with the adverse effect of oxidized GSSG, which elevates the redox potential (65). Alternative reducing agents like *L*-Cys and DTT, with comparable redox potentials of approximately −220 mV (66) or −330 mV (67), respectively, also preserved maximal and long-term Hox activity after oxygen exposure (Fig. 7). Notably, despite DTT having the lowest redox potential (68), *L*-Cys, with the most positive redox potential, exhibited the strongest protective effect, followed by GSH (redox potential of GSH is −240 mV (69)) (Fig. 7). This indicates that Hox activity recovery post-oxygen exposure *in vitro* is not solely influenced by the redox potential. This can also be the case *in vivo* in concert with other factors, such as posttranslational modifications (68, 70).

Direct interactions between GSH (other thiol compounds) and Hox could also contribute to the protective effect. Previous studies have shown that GSH and *L*-Cys modulate hydrogenase activity either negatively (71) or positively (72). In our study, GSH exhibited a beneficial effect on Hox activity within a concentration range of 2.5 to 10 mM, followed by a reduction in enzymatic activity at higher concentrations (Fig. 5). A direct interaction between GSH and Hox of *Synechocystis* has been suggested due to a potential posttranslational glutathionylation site at Cys_289_ in HoxF (30), which could influence enzymatic activity (23) and oligomerization (73, 74). However, since similar protective effects were observed with other thiol-containing compounds, the mechanism does not appear to be GSH-specific, arguing against posttranslational glutathionylation. Identifying posttranslational modifications was beyond the scope of this study and should be addressed in future research.

The sensitivity of [NiFe] hydrogenases to oxygen is commonly attributed to its interaction with the [NiFe] active site, leading to the formation of inactivated Ni-states, Ni-A and Ni-B (43). Using BN-PAGE and in-gel activity staining, we observed that oxygen exposure alters Hox complex integrity and promotes the formation of distinct subcomplexes (Fig. 3). Oxygen exposure resulted in a shift in the in-gel activity staining pattern (Fig. 3*A*), indicating the disintegration of the holoenzyme Hox (HYUFE)_2_ complex into smaller subcomplexes. However, GSH supplementation did not alter the staining pattern (Fig. 3, *A* and *B*). The distribution of Hox subunits across the BN gel lane remained largely unchanged after oxygen exposure, even with GSH addition (Fig. 3*C*). These findings argue against a significant role of GSH in Hox complex restoration. Nonetheless, the effect of GSH in photometric assays was observed only after a 2-h incubation, suggesting that the incubation time for reactivation before gel electrophoresis (which was a few minutes) might have been insufficient for reactivation to become evident on BN-PAGE. In *Escherichia coli*, dimerization of the oxygen-insensitive [NiFe] hydrogenase Hyd-1 enhances BV-dependent H_2_ oxidation activities twofold and improves reactivation after oxygen exposure, likely facilitated by electrons from [FeS] clusters of other subunits (58). Given that Hox also forms a dimer *in vivo* (39), similar mechanisms may underlie Hox reactivation after oxygen exposure. However, this is beyond the scope of our study and warrants further investigation.

Determining H_2_-driven BV reduction rates is a valuable *in vitro* activity assay to follow hydrogenase activity. Yet, our study revealed that different Hox complexes exhibit varying H_2_-oxidizing activities (Fig. 2). This variation likely depends on the accessibility of BV from the protein surface to the [FeS] clusters, which facilitate electron conduction. Due to its bulkiness and MM of 409.35 g/mol, BV is unlikely to reach the active site of Hox in *Synechocystis*. This aligns with previous molecular dynamics simulations indicating that only molecules with a MM below 131.29 g/mol can access hydrogenase active centers through hydrophobic channels (75). Our calculations of the tunnels in Hox reveal that the active site in HoxH is accessible only through a narrow tunnel, not exceeding 2 Å in diameter, making it impassable for bulky viologens (Fig. S6). This suggests that BV primarily extracts electrons from one of the [FeS] clusters of other Hox subunits at the protein surface, rather than being reduced at the HoxH active center, indicating that different Hox subcomplexes might exhibit different H_2_-driven BV reduction activities depending on the accessibility of BV to the extracted electrons (Fig. S6). If this holds true, coupling or interacting different active Hox submodules (HoxHY, HoxHYE, or HoxHYEU) with other electron-accepting or electron-donating proteins is feasible. Efficient electron transfer should be possible if the redox potentials of the electron acceptor and donor pairs are compatible and the distances between the interacting [FeS] clusters are less than 14 Å (55). This has been demonstrated in previous studies, where HoxHY was coupled to photosystem I for photosynthetic H_2_ production (76, 77), showcasing that coupling of hydrogenases to other proteins for biotechnological applications might be also feasible.

Taken together, our findings demonstrate that both GSH and GSSG influence H_2_ oxidation rates of Hox from *Synechocystis in vitro*. While GSH and similar thiol-containing compounds promote reactivation after exposure to air, GSSG has an inhibitory effect. The protective and restorative impact of thiol-containing compounds like GSH, *L*-Cys, and DTT on Hox activity may primarily arise from two main beneficial effects: scavenging oxygen in the enzyme’s active site and modulating the redox potential. However, we cannot rule out posttranslational modifications of cysteine residues in Hox, which is within the scope of our future study. Our *in vitro* experiments suggest that the regulation of Hox activity *in vivo*, with or without oxygen, involves intracellular redox potentials, primarily influenced by the GSH/GSSG pool, as suggested also previously (49). Additionally, other reduced cysteine residues, the GSH precursor γ-glutamyl cysteine, and other antioxidants such as ophthalmate, ergothioneine, and hercynine (25), as well as the GSH-ascorbate cycle (23), could contribute to redox environment modulation *in vivo*, thereby protecting Hox activity. Engineering intracellular redox potential through genetic and cultivation methods could offer a novel approach to optimizing photoautotrophic H_2_ production. Thiol-containing reducing agents may also hold promise for *in vitro* applications involving oxygen-sensitive enzymes due to their protective effects. Thus, our study may have implications for the regulation and reactivation of other oxygen-sensitive redox enzymes, warranting investigation in future studies.

## Experimental procedures

### Chemicals and microbial strains

Two *Synechocystis* strains were used in this study: (*i*) the PCC6803 WT strain and (*ii*) a *Synechocystis* strain deficient in the octacistronic *hox* operon, referred to as Δ*hox* strain in the following. This strain had its ORF of *hox* replaced by a kanamycin resistance gene, as described elsewhere (78). Chemicals used in this study were purchased at the highest available purity, mainly sourced from Sigma-Aldrich unless otherwise specified. The separation of protein complexes was conducted *via* BN-PAGE using precast 4 to 16% gradient Bis-Tris native gels (NativePAGE Novex, Invitrogen), along with a NativeMark Unstained Protein Standard (Invitrogen) and the Novex Tris-Glycine Native sample buffer at a 4-fold concentration (Invitrogen). The trypsin digestion enzyme used for nano-liquid chromatography-tandem mass spectrometry (nLC-MS/MS) sample preparation, was procured from Promega, and all chemicals used for sample preparation and nLC-MS/MS analysis were of LC-MS grade. ZipTip-μC_18_ tips (Merck Millipore) were used to desalt the peptide samples prior to nLC-MS/MS analysis.

### Cultivation and cell harvesting

*Synechocystis* sp. PCC6803 and Δ*hox* strains were cultivated in BG11 medium supplemented with 10 mM Hepes (pH 8.0) in a Labfors rotary shaker (INFORS HT) under controlled conditions: irradiance of 50 μmol photons m^−2^ s^−1^, agitation at 120 rpm, and a temperature of 30 °C, maintaining ambient CO_2_ concentrations. The composition of the BG11 medium, as provided by van Alphen *et a*l. (79), was modified by the replacement of carbonate with 50 mM NaHCO_3_, an increase in the concentration of Na_2_EDTA to 18 μM, and FeCl_3_ x 6 H_2_O to 6 μM. For the cultivation of Δ*hox* strain, 50 μg/ml kanamycin was added to the medium. To enhance hydrogenase gene expression, cells were grown under nitrogen limitation (Fig. S1), which was achieved by inoculating BG11 medium with reduced nitrate concentration of 1 mM to a starting A_750_ of 0.1 using a preculture grown in standard BG11 medium (resulting in an approximate dilution of 1:50). Cells were subsequently cultivated for 8 days and harvested by centrifugation at 5000*g* for 5 min at 20 °C. Pellets obtained from 15- or 50-mL culture were stored at −20 °C.

### Cell disruption and preparation of anaerobic crude extracts

All steps were conducted within a Coy anaerobic chamber under a nitrogen atmosphere containing 2.5 to 5% hydrogen (Coy Laboratory Products). Frozen cell pellets were introduced into the anaerobic chamber and resuspended in anoxic buffer A, composed of 50 mM phosphate buffer supplemented with 10% (v/v) glycerol (pH 6.8), prior to cell disruption through bead beating using 0.1 mm zirconia beads at 6 m/s for 30 s, in two cycles, *via* a Savant Bio101 FastPrep FP120 cell disruptor (Thermo Fisher Scientific). Subsequent to disruption, cellular debris and undisrupted cells were removed by centrifugation at 11,000*g* and 4 °C for 5 min. The supernatant underwent a second centrifugation step at 16,700*g* and 4 °C for 45 min. To investigate the stabilizing effect of, for example, glutathione on the hydrogenase activity, the supernatant from the last step containing the protein crude extract was manually desalted within the anaerobic chamber using a 5 ml HiTRAP desalting column (Cytiva) following the ‘syringe protocol’ provided by the supplier. This desalting step facilitated the separation of low-molecular components, including cofactors and reducing agents, from the proteins. In brief, the column was equilibrated with five column volumes (25 ml) of anoxic buffer A before applying 0.5 ml of the *Synechocystis* crude extract. Proteins were subsequently eluted in 2.5 ml buffer A. Protein concentration was estimated by measuring absorbance at 280 nm using DeNovis DS-11 FX + spectrophotometer and assuming that 1 absorbance unit (AU) corresponded to 1 mg/ml protein.

### Photometric determination of hydrogenase activity

Hox hydrogenase enzyme activity (H_2_-dependent reduction of BV) in crude extracts of *Synechocystis* was determined in the anaerobic chamber under defined gas mixture containing approximately 3.6% hydrogen (H_2_), measuring the absorbance increase at 545 nm. As photometric enzyme assays were performed in a 96-well quartz plate covered with PE foil (Kisker Biotech GmbH) using the BioTek Synergy HT photometer (Agilent Technologies), rather than in a standardized 1-cm quartz cuvette, the experimental determination of the extinction coefficient of reduced BV at 545 nm (ε_545_) was necessitated. Therefore, defined concentrations of BV were fully reduced by titanium(III)citrate. The absorbance maxima at 545 nm were divided by the BV concentration, resulting in ε′_545_. The ε_545_ value of BV was then normalized to the optical path length of the 96-well plate, with an ε′_545_ of 6540 M^−1^.

Prior setting up hydrogenase activity assays, desalted protein extracts from *Synechocystis* were diluted in buffer A, supplemented with varying concentrations of reducing agents such as glutathione, *L*-cysteine, or DTT, achieving a final protein concentration of 0.5 mg/ml. Subsequently, three replicates per condition, each containing 100 μl aliquots in Eppendorf tubes with perforated lids, allowing for the measurement of three replicates per tube, were exposed to oxygen by gently shaking the tubes in air for 3 to 5 s. A volume of 20 μl of each protein sample, whether exposed or not to oxygen, was pipetted into a 96-well quartz plate, and the reaction was initiated by adding 200 μl of the activity mix, resulting in a final concentration of 1 mM BV in buffer A. Typically nine technical replicates, unless otherwise specified, were set up for each condition. NEC were prepared, containing buffer instead of desalted protein extracts. Furthermore, the background activity of H_2_-dependent BV reduction, stemming from proteins in *Synechocystis* not affiliated with the Hox hydrogenase, was determined by using equal protein amounts of crude extracts from the Δ*hox* strain. The photometric assay was conducted at 30 °C for 200 min, with absorbance readings taken at 545 nm every 3 min. In the process of data analysis, the absorbances of NEC and/or the background activities originating from the Δ*hox* strain were subtracted from the experimental absorbances derived from setups with *Synechocystis* crude extracts after various treatments. From the corrected slopes, the change in BV concentration was determined, followed by the calculation of the enzyme activity in units (U), representing a conversion of 1 μmol/min H_2_ or BV, which was normalized to 1 mg of protein. To comprehensively capture the kinetics of H_2_-driven BV reduction and the impact of oxygen exposure on the enzyme, three parameters were calculated: (*i*) the initial hydrogenase activity observed in the first 30 min of measurement, (*ii*) the maximal activity, defined as the highest reduction rate for BV within any 30-min time window; (*iii*) the long-term activity, defined as the enzyme’s activity between 120 and 150 min of incubation; and (*iv*) the residual maximal hydrogenase activity after oxygen exposure, determined by comparing it with the enzyme activity in the same buffer without exposure to oxygen. The rates calculated from kinetic measurements are listed in Supporting Information ‘Table S1’. All data underwent comprehensive statistical analysis as detailed in the Supporting Information, section ‘1.1 Statistical evaluation’.

### BN-PAGE and in-gel hydrogenase activity staining

To separate protein complexes of *Synechocystis*, BN-PAGE was conducted under anoxic and chilled conditions within the anaerobic chamber using precast 4 to 16% gradient gels and the NativeMark Unstained Protein Standard (Invitrogen). Electrophoresis buffers for the light blue gels were prepared following the manufacturer’s instructions, anaerobized by nitrogen gas flushing for approximately 30 min, and supplemented with 1 mM DTT. Gels and buffers were introduced into the anaerobic chamber at least 1 day before the electrophoresis. Prior to loading protein samples onto the gel, a pre-run of approximately 30 min was conducted at 150 V to flush the gels with anaerobic buffers. After the pre-run, sample wells were loaded with 200 or 400 μg of *Synechocystis* proteins, amended with approximately 0.05% (w/v) Coomassie G-250 sample additive (Invitrogen), before electrophoresis was run at a constant current of 7 to 9 amperes for approximately 3.5 h. When samples exposed to oxygen were subjected to BN-PAGE, they were exposed to air for 30 min at 4 °C and 300 rpm before electrophoresis. Cooling pads surrounded the gel chamber during the run to protect enzymes and preserve enzymatic activity. After electrophoresis, the gel was subjected to in-gel hydrogenase staining as detailed in the Supporting Information, section ‘1.2 In-gel hydrogenase activity staining’.

### Protein mass spectrometry

For complexome analysis, stained BN PAGE triplicate lanes were cut into 10 slices (Fig. 3, *A* and *B*) using a razor blade that was cleaned with ethanol after each cut. Additionally, the gel bands from hydrogenase activity–stained gels (Fig. 2*A*) were also sliced. These gel slices and bands were prepared for nLC-MS/MS analysis through destaining, reduction of disulfide bridges, subsequent cysteine carbamidylation and tryptic in-gel digestion, following established protocol (51). The resulting peptides were extracted in multiple steps using 50% (v/v) acetonitrile, 0.1%(v/v) formic acid, or 5 mM ammonium bicarbonate and subsequently desalted using ZipTip-μC_18_ material (Merck Millipore). After desalting, peptides were dried in the vacuum centrifuge and resuspended in 20 μl of 0.1% (v/v) formic acid before mass spectrometry on the Orbitrap Fusion Tribrid mass spectrometer (Thermo Fisher Scientific) coupled to a nLC system Dionex Ultimate 3000RSLC (Thermo Fisher Scientific) *via* an electrospray ion source TriVersa NanoMate (Advion). Peptides (injection volume 5 μl) were separated on an Acclaim PepMap100 C_18_ column (Thermo Fisher Scientific) at a flow rate of 0.3 μl/min, setting up parameters described previously (80). Subsequently, proteins were identified using Proteome Discoverer v2.2 (Thermo Fisher Scientific) as described elsewhere (80). Detailed information of protein identification is given in the Supporting Information, section ‘1.3 Protein identification’.

### Bioinformatics and protein structure prediction

Amino acid sequences for Hox hydrogenase subunits from *Synechocystis* sp. PCC6803 were obtained from the National Center for Biotechnology Information (NCBI) Database (81). These sequences were used for structure prediction of the dodecameric Hox(HYEUF)_2_ complex using AlphaFold 2 ColabFold multimer platform, with default settings (82). Refinements of the top-ranked *in silico* structure were performed with PyMol (https://www.pymol.org/support.html?). Cofactors were positioned by superimposing a pentameric Hox(HYEUF) structure with the experimentally determined structure of the homologous soluble NAD^+^-dependent hydrogenase (PDB: 5XF9) of *H. thermoluteolus* TH-1 to determine the coordinating cysteine residues before transferring the [FeS] clusters at the same position in the dimeric complex. The CAVER 3.0 PyMol plugin, with default settings, was employed to identify tunnels and cavities within the proteins (83).

## Data availability

Data described in the manuscript are contained within it. Raw data is available upon request to Andreas Schmid at andreas.schmid@ufz.de.

## Supporting information

This article contains supporting information.

## Conflict of interest

The authors declare that they have no conflicts of interest with the contents of this article.
