# Supplementary material for: Reactivation and long-term stabilization of the [NiFe] Hox hydrogenase of Synechocystis sp. PCC6803 by glutathione after oxygen exposure
Source: J Biol Chem. 2024 Dec 14;301(1):108086. doi: 10.1016/j.jbc.2024.108086 (PMC11780932; doi:10.1016/j.jbc.2024.108086)
Supplement: Supporting information [file mmc1.pdf]

Supporting Information to the Manuscript:

Reactivation and long-term stabilization of the [NiFe] Hox hydrogenase of *Synechocystis* sp. PCC6803 by glutathione after oxygen exposure

Merle Romig[1], Marie Eberwein[2], Darja Deobald[2*], Andreas Schmid[1*]

[1]Department of Solar Materials, Helmholtz Centre for Environmental Research - UFZ GmbH, Permoserstrasse 15, 04318 Leipzig, Germany

[2]Department of Molecular Environmental Biotechnology, Helmholtz Centre for Environmental Research - UFZ GmbH, Permoserstrasse 15, 04318 Leipzig, Germany

1 Supplementary experimental procedures

1.1 Statistical evaluations

Standard deviations were calculated for each set of technical replicate measurements. Statistical significance was assessed based on the distribution of the data, which was determined using the Shapiro-Wilk test. If the data set showed normal distribution, the bivariable Student's T-test was employed. For F-test with p-values above 0.05, equal variance was assumed. When normal distribution was not confirmed by the Shapiro-Wilk test, the Mann-Whitney U-test was applied. P-values greater than 0.05 were considered nonsignificant, while $p < 0.05$ was indicated by *, $p < 0.01$ by **, and $p < 0.001$ by ***.

1.2 In-gel hydrogenase activity staining

Post-electrophoresis, the gel was washed with 50 mM phosphate buffer (pH 6.8) supplemented with 10 % (v/v) glycerol and 10 mM *L*-cysteine. Replicate lanes, each containing cell extracts from individual conditions along with the protein ladder, were separated from the remainder of the gel and stained using a 1-fold Coomassie Brilliant Blue R-250 staining solution, consisting of 10 % (v/v) ethanol, 2 % (v/v) acetic acid, and 0.02 % (w/v) Coomassie R-250. Therefore, the gel was washed with water, followed by coverage with the staining solution, heating in a microwave, and a 20-minute incubation. Excess staining was removed through multiple washing steps with

water utilizing microwave-assisted heating. Unstained gel lanes were subjected to either complexome analysis by nano-liquid chromatography-tandem mass spectrometry (nLC-MS/MS), as described in the subsequent chapter, or a hydrogenase activity staining procedure.

Hydrogenase activity staining was conducted in sealed bottles using 50 mM phosphate buffer, 10 % (v/v) glycerol, 10 mM *L*-cysteine (pH 6.8), supplemented with 5 mM 2,3,5-triphenyltetrazolium chloride and 1 mM BV or methyl viologen, respectively. Hydrogenase staining was conducted under two conditions: *(i)* utilizing 100 % H_2 atmosphere and benzyl viologen or methyl viologen as artificial electron acceptor, *(ii)* or using 3.6 % H_2 atmosphere from the anoxic chamber and benzyl viologen as electron acceptor. The gels were incubated overnight at RT.

1.3 Protein identification

Proteome Discoverer v2.2 (Thermo Scientific) was used for protein identification based on the *Synechocystis* sp. PCC6803 proteome from UniProt database (taxonomy ID: 1148) through the SequestHT search engine. The following parameters were set: carbamidomethylation of cysteine residues was set as fixed, while the oxidation of methionine was selected as a dynamic modification; trypsin cleavage sites were selected, allowing for a maximum of two cleavage sites missed per protein; precursor and fragment mass tolerances were set to 3 ppm and 0.6 Da, respectively. Stringent criteria were applied with a false discovery rate threshold of 1 % for peptide identification using the Target Decoy PSM Validator node. The Minora node in Proteome Discoverer facilitated protein quantification through label-free quantification based on MS1 precursor intensity values. Relative protein abundance was calculated from the ratio of a protein’s abundance in the specific gel slice normalized to its overall abundance across all slices within a given BN-PAGE gel lane. Only proteins detected with high confidence in false discovery rate were included in the analysis.

2 Supplemetary figures

Subunit / Gel band	MS1 intensity (Relative abundance)				
	**HoxH**	**HoxY**	**HoxU**	**HoxF**	**HoxE**
**1**	3.1×10^5 (0.04%)	/	/	/	/
**2**	1.4×10^6 (0.05%)	/	2.5×10^6 (0.09%)	3.7×10^5 (0.01%)	/
**3**	1.2×10^7 (0.18%)	6.9×10^5 (0.01%)	1.9×10^7 (0.29%)	6.6×10^6 (0.10%)	6.0×10^5 (0.01%)
**4**	1.4×10^7 (0.16%)	3.4×10^5 (0.00%)	2.6×10^7 (0.30%)	1.1×10^7 (0.13%)	1.9×10^6 (0.02%)
**5**	2.5×10^7 (0.23%)	3.9×10^6 (0.04%)	4.9×10^7 (0.46%)	2.1×10^7 (0.20%)	1.0×10^6 (0.01%)
**6**	2.9×10^6 (0.13%)	/	6.3×10^6 (0.29%)	2.0×10^6 (0.09%)	5.2×10^5 (0.02%)
**7**	7.7×10^6 (0.21%)	/	3.5×10^7 (0.93%)	8.4×10^6 (0.22%)	6.1×10^5 (0.02%)
**8**	5.2×10^6 (0.22%)	/	4.6×10^6 (0.19 %)	3.1×10^5 (0.01%)	/
**9**	3.3×10^7 (1.18%)	3.4×10^5 (0.01%)	2.5×10^7 (0.92%)	1.8×10^6 (0.07%)	5.6×10^5 (0.02%)

Figure S1. Protein mass spectrometry (nLC-MS/MS) of activity stained gel bands sliced from a Blue Native PAGE, which was used to separate extracts from *Synechocystis* sp. PCC6803. Hox hydrogenase subunits identified in the gel bands *via* nLC-MS/MS, with a minimum of two unique peptides. MS1 intensities represent the summed intensities of peptides derived from the same protein, providing an overall estimation of the protein's abundance without offering absolute quantification. The relative abundance (%) of each subunit within the gel slice was determined by normalizing the MS1 intensities of each subunit to the total MS1 intensities of all proteins detected within that gel band.

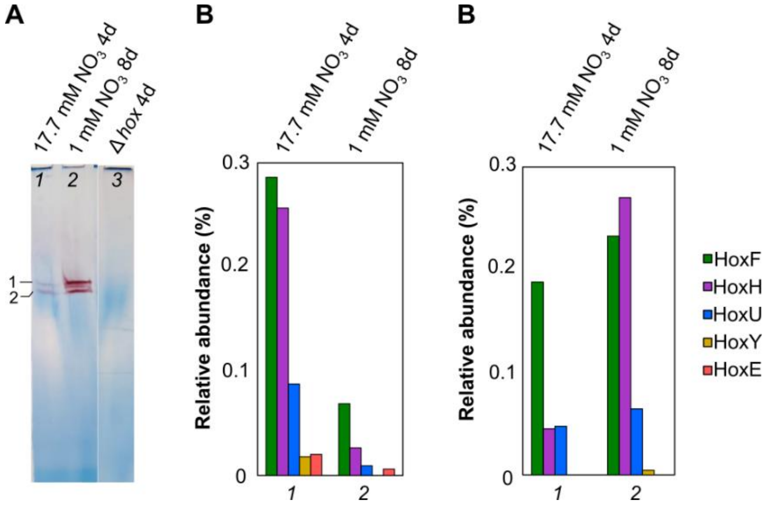

Figure S2. Protein crude extracts (200 µg) from *Synechocystis* sp. PCC6803 wildtype and Hox-deficient (Δ*hox*) strains separated using Blue Native (BN)-PAGE, followed by in-gel hydrogenase activity staining and analysis of active bands (1-2) *via* mass spectrometry (nLC-MS/MS). (A) In-gel activity staining performed on crude extracts from *Synechocystis* wildtype cultivated for 4 days under nitrogen-rich conditions (lane *1*, 17.7 mM NO_3) and for 8 days under nitrogen-depleted conditions (lane *2*, 1 mM NO_3), as well as on the Δ*hox* strain cultivated for 4 days (lane *3,* 17.7 mM NO_3). Activity staining was performed using benzyl viologen as the electron acceptor and approximately 3.6 % hydrogen as the electron donor. Active gel bands 1 and 2 were analyzed *via* nLC-MS/MS. **(B)** Hox hydrogenase subunits identified within the active gel band 1, and **(C)** within the active gel band 2 from gel lanes *1* and *2*, respectively, were determined through nLC-MS/MS. The relative abundance (%) of each subunit within the individual gel slice was calculated by normalizing the MS1 intensity of each subunit to the total MS1 intensities of all proteins detected within the gel band.

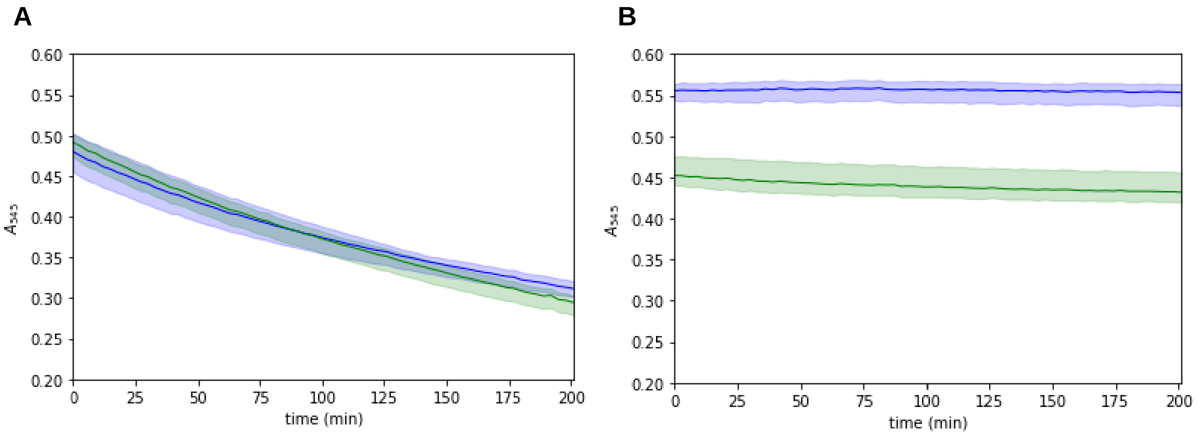

Figure S3. Negative controls implemented to assess the background activity associated with benzyl viologen (BV) re-oxidation, as indicated by absorbance decrease at 545 nm (A_{545}), during the photometric Hox activity assays. (A) BV re-oxidation background activity not related to Hox hydrogenase was evaluated using crude extracts from Hox-deficient (Δ*hox*) *Synechocystis* sp. PCC6803 strain, with glutathione (GSH) (green line) or without GSH addition (blue line). **(B)** Abiotic control experiments, conducted in the absence of *Synechocystis'* crude extracts, examined the stability of reduced BV in buffer, both with GSH (green line) and without GSH (blue line).

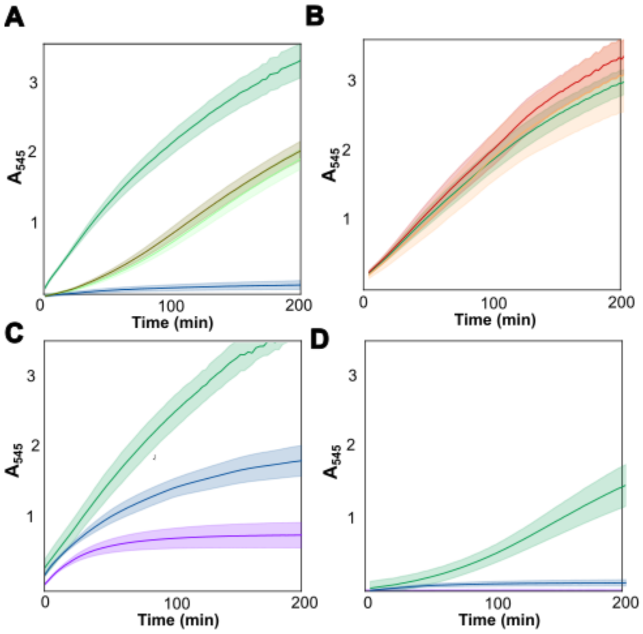

Figure S4. Hox activity from protein crude extracts of *Synechocystis* sp. PCC6803, monitored through an absorbance increase at 545nm (A_{545}). (A) Changes in A_{545} in anoxic protein extract samples with 2.5 mM glutathione (GSH) (dark green line), samples exposed to oxygen without GSH (blue line), and with 2.5 mM GSH added before (light green line) or after oxygen exposure (olive line). **(B)** Changes in A_{545} in anoxic protein extracts with 2.5 mM GSH (green line), 2.5 mM *L*-cysteine (red line), or 1.25 mM dithionite (DTT) (yellow line). **(C)** Changes in A_{545} in anoxic protein extracts with 2.5 mM GSH (green line), 1.25 mM oxidized glutathione (GSSG) (violet line), and without supplements (blue line). **(D)** Changes in A_{545} in oxygen-exposed samples with 2.5 mM GSH (green line), 1.25 mM GSSG (violet line), and without any additives (blue line).

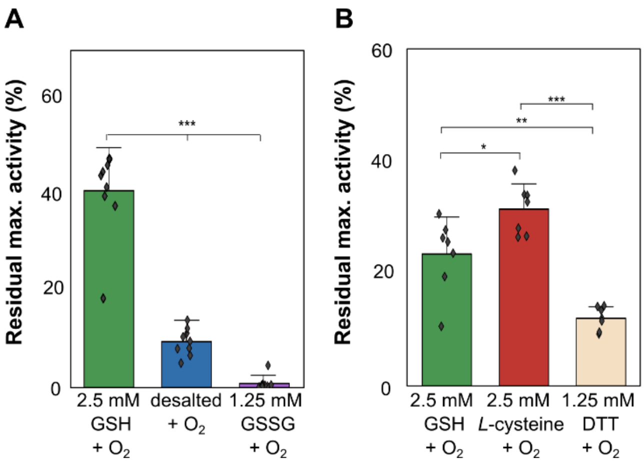

Figure S5. Residual Hox activity in cell-free extracts after exposure to oxygen (O_2) for 3 – 5 seconds. Residual activities were calculated by normalizing the maximal activity of oxygen-exposed samples to their anoxic activities under the same conditions. Bar charts represent average values, with individual data points shown as diamonds and standard deviations as error bars. **(A)** Residual maximal activities of samples after oxygen exposure, supplemented with either reduced glutathione (GSH, green) or the oxidized glutathione (GSSG, violet), as well as samples without additives (blue). **(B)** Residual maximal activities of samples exposed to O_2 and supplemented with GSH (green), *L*-cysteine (red) or DTT (beige). Statistical significance was assessed using an unpaired Student's t-test for normally distributed data sets and a Mann-Whitney U-test for non-normally distributed samples, with *: $p < 0.05$, **: $p < 0.01$, and ***: $p < 0.001$.

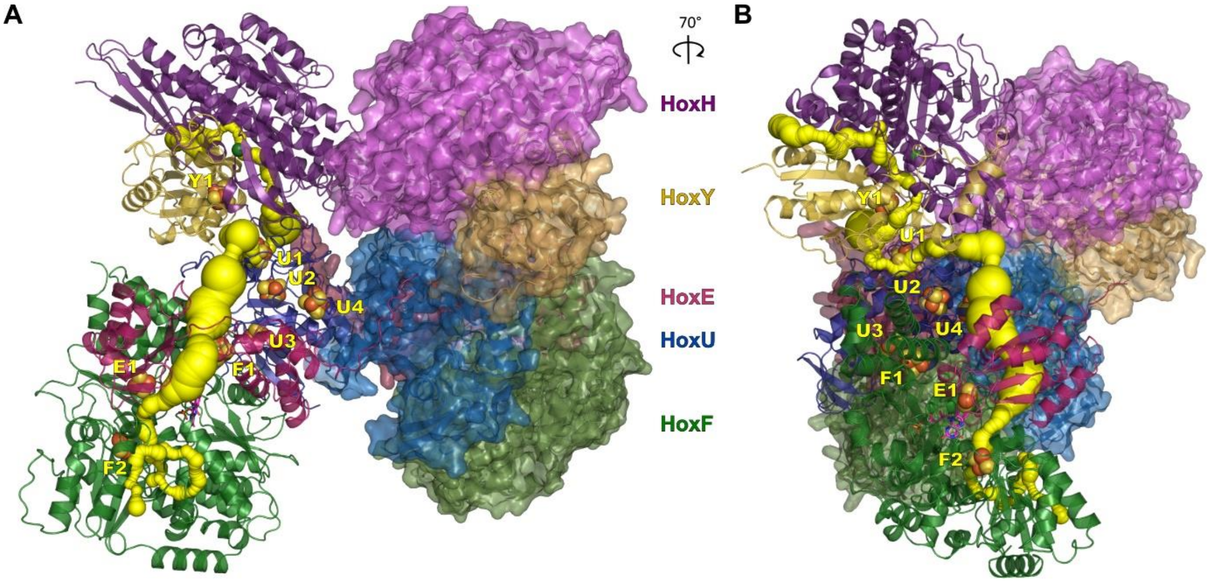

Figure S6. ***In-silico*** **predicted structure of the Hox(HYEUF)$_2$ complex from** ***Synechocystis*** **sp. PCC6803, calculated using AlphaFold 2.** The predicted water-filled tunnel spanning the Hox complex, calculated with CAVER 3.0, is shown in yellow spheres. Tunnels with a minimal radius of approximately 0.9 Å are depicted. The iron-sulfur cofactors and FMN, forming an electron-conducting 'wire' are shown as orange-yellow spheres and magenta sticks. **(A)** On the left side, the heteropentamer HoxHYEUF is in cartoon representation, with predicted iron-sulfur clusters (Y1 to F2, yellow-orange spheres) and FMN (pink, stick representation), while the second HoxHYEUF pentamer on the right side is in surface representation. **(B)** The Hox(HYEUF)$_2$ structure rotated 70° to the right around the y-axis.

3 Supplemetary tables

Table S1. Summary of all Hox activities, measured in this study, by tracking the absorbance changes at 545 nm originating from hydrogen-driven benzyl viologen (BV) reduction. Crude extracts of *Synechocystis* sp. PCC6803 were either exposed (+) or not exposed (-) to oxygen (O_2) for 3 – 5 seconds. Initial Hox activity was determined within the first 30 minutes of incubation, while maximal (max.) Hox activity was the highest activity observed during any 30-minute interval. Long-term activity was calculated within the 120 – 150-minute incubation window. Abbreviations: GSH – reduced glutathione, GSSG – oxidized glutathione, *L*-Cys – *L*-cysteine, DTT – dithiothreitol.

**Results' section**	**Condition/Additive**	**O_2**	**Initial activity**	**Max. activity**	**Long-term activity**	***n***
			**(U/$g_{protein}$)**			
**3.4**	not desalted/-	-	19.0 ± 2.4	19.0 ± 2.4	0.1 ± 0.4	9
	not desalted/-	+	0.9 ± 0.2	0.9 ± 0.2	-0.1 ± 0.0	9
	desalted/-	-	30.1 ± 2.4	30.1 ± 2.4	7.8 ± 2.8	9
	desalted/-	+	2.9 ± 0.8	2.9 ± 0.8	0.2 ± 0.2	9
	desalted/2.5 mM GSH	-	40.2 ± 2.5	41.2 ± 2.3	26.3 ± 1.4	9
	desalted/2.5 mM GSH	+	4.7 ± 0.7	16.8 ± 3.5	16.6 ± 3.6	9
	desalted/2.5 mM GSH	-	40.6 ± 3.0	41.1 ± 2.9	20.7 ± 1.7	9
	desalted/2.5 mM GSH before O_2 exposure	+	6.9 ± 0.8	20.6 ± 0.8	20.5 ± 1.5	9
	desalted/2.5 mM GSH after O_2 exposure	+	8.6 ± 1.0	21.3 ± 1.4	20.3 ± 1.1	9
	desalted/-	+	2.9 ± 0.8	2.9 ± 0.7	0.7 ± 0.4	9
**3.5**	desalted/2.5 mM GSH	-	22.6 ± 1.7	26.4 ± 3.6	21.4 ± 2.2	8
	desalted/2.5 mM GSH	+	2.1 ± 0.3	7.4 ± 1.8	6.3 ± 1.1	8
	desalted/4.5 mM GSH	-	20.0 ± 1.9	23.9 ± 1.5	19.8 ± 0.9	8
	desalted/4.5 mM GSH	+	2.2 ± 0.4	9.4 ± 2.1	7.5 ± 1.7	8
	desalted/10 mM GSH	-	4.4 ± 0.9	21.6 ± 3.1	19.7 ± 2.7	9
	desalted/10 mM GSH	+	0.6 ± 0.1	12.7 ± 3.0	6.2 ± 3.0	8
	desalted/-	-	30.1 ± 2.4	30.1 ± 2.4	7.8 ± 2.8	9
	desalted/-	+	2.9 ± 0.8	2.9 ± 0.8	0.2 ± 0.2	9
	desalted/2.5 mM GSH	-	40.2 ± 2.5	41.2 ± 2.3	26.3 ± 1.4	9
	desalted/2.5 mM GSH	+	4.7 ± 0.7	16.8 ± 0.7	16.6 ± 3.6	9
	desalted/1.25 mM GSSG	-	23.0 ± 3.1	23.0 ± 3.1	0.9 ± 0.7	9
	desalted/1.25 mM GSSG	+	0.1 ± 0.3	0.2 ± 0.3	0.0 ± 0.1	9
**3.6**	desalted/2.5 mM *L*-Cys	-	29.8 ± 4.2	33.3 ± 3.5	22.8 ± 4.3	7
	desalted/2.5 mM *L*-Cys	+	4.5 ± 0.9	10.5 ± 1.94	10.2 ± 1.6	7
	desalted/1.25 mM DTT	-	26.8 ± 5.4	34.8 ± 5.7	19.0 ± 3.2	7
	desalted/1.25 mM DTT	+	2.4 ± 0.5	4.2 ± 0.7	3.9 ± 0.6	7
	desalted/2.5 mM GSH	-	28.1 ± 4.1	30.7 ± 4.3	19.0 ± 1.8	7
	desalted/2.5 mM GSH	+	2.7 ± 0.9	7.2 ± 1.9	6.9 ± 1.9	7
